# Genome-wide identification, molecular evolution, and expression analysis provide new insights into the APETALA2/ethylene responsive factor (AP2/ERF) superfamily in *Dimocarpus longan* Lour

**DOI:** 10.1186/s12864-020-6469-4

**Published:** 2020-01-20

**Authors:** Shuting Zhang, Chen Zhu, Yumeng Lyu, Yan Chen, Zihao Zhang, Zhongxiong Lai, Yuling Lin

**Affiliations:** Institute of Horticultural Biotechnology, Fujian Agriculture and Forestry University, Fuzhou, 350002 Fujian China

**Keywords:** *Dimocarpus longan*, AP2/ERF, Single nucleotide polymorphisms, Insertions and deletions, Alternative splicing events, Expression patterns

## Abstract

**Background:**

The APETALA2/ethylene responsive factor (AP2/ERF) superfamily members are transcription factors that regulate diverse developmental processes and stress responses in plants. They have been identified in many plants. However, little is known about the AP2/ERF superfamily in longan (*Dimocarpus longan* Lour.), which is an important tropical/subtropical evergreen fruit tree that produces a variety of bioactive compounds with rich nutritional and medicinal value. We conducted a genome-wide analysis of the AP2/ERF superfamily and its roles in somatic embryogenesis (SE) and developmental processes in longan.

**Results:**

A genome-wide survey of the AP2/ERF superfamily was carried out to discover its evolution and function in longan. We identified 125 longan *AP2/ERF* genes and classified them into the ERF (101 members), AP2 (19 members), RAV (four members) families, and one Soloist. The *AP2* and Soloist genes contained one to ten introns, whereas 87 genes in the ERF and RAV families had no introns. Hormone signaling molecules such as methyl jasmonate (MeJA), abscisic acid (ABA), gibberellin, auxin, and salicylic acid (SA), and stress response *cis*-acting element low-temperature (55) and defense (49) boxes also were identified. We detected diverse single nucleotide polymorphisms (SNPs) between the ‘Hong He Zi’ (HHZ) and ‘SI JI MI’ (SJM) cultivars. The number of insertions and deletions (InDels) was far fewer than SNPs. The AP2 family members exhibited more alternative splicing (AS) events in different developmental processes of longan than members of the other families. Expression pattern analysis revealed that some AP2/ERF members regulated early SE and developmental processes in longan seed, root, and flower, and responded to exogenous hormones such as MeJA, SA, and ABA, and 2,4-D, a synthetic auxin. Protein interaction predictions indicated that the Baby Boom (BBM) transcription factor, which was up-regulated at the transcriptional level in early SE, may interact with the LALF/AGL15 network.

**Conclusions:**

The comprehensive analysis of molecular evolution and expression patterns suggested that the AP2/ERF superfamily may plays an important role in longan, especially in early SE, and in seed, root, flower, and young fruit. This systematic analysis provides a foundation for further functional characterization of the AP2/ERF superfamily with the aim of longan improvement.

## Background

The AP2/ERF superfamily is one of the largest transcription factor families in plants. The most prominent feature of the AP2/ERF superfamily is that all its members contain at least one AP2 conserved domain, and depending on the number of AP2 domains, the AP2/ERF superfamily has been divided into the ERF, AP2, RAV families, and one Soloist [1]. The ERF family contains the most members, each of which has a single AP2 domain. Sakuma et al. [2] divided the ERF family into two subfamilies, ERF and DREB, on the basis of differences in the sequences of their DNA-binding domains [2]. Nakano et al. [1] analyzed the phylogeny and conserved domains of the ERF family in *Arabidopsis* and rice and divided them into 12 and 15 groups, respectively [1]. Generally, the ERF subfamily members bind to an AGCCGCC sequence [3] and the DREB subfamily members typically interact with a CCGAC core sequence [4]. The AP2 family members contain two AP2 domains, bind to an GCAC(A/G)N(A/T)TCCC(A/G)ANG(C/T) sequence, and can be divided into AP2 and AIL subfamilies according to the phylogeny. The RAV family members contain one AP2 domain and a B3 domain, which is a DNA-binding domain conserved in other families, and bind to CAACA and CACCTG sequences [5]. The Soloist gene usually contain a single AP2 domain, which is usually diverged from other families.

The functions of the AP2/ERF superfamily members have been studied widely in plants and are known to regulate diverse developmental processes and stress responses. ERF subfamily members, which bind to the GCC-box, are involved in signaling pathways during abiotic and biotic stress responses as well as in pathways related to plant development, such as hormone signaling pathways [6–8], the oxygen sensing pathway [9], and nutrition signaling pathway [10]. Moreover, several ERF subfamily members have been reported to play vital roles in metabolite biosynthesis [11]. DREB subfamily members bind to the dehydration-responsive element/C-repeat (DRE/CRT) and are considered to the main regulators of plant abiotic stresses, including drought [12], cold [13], heat [14], and salt stress [15]. Several DREB subfamily genes were also reported to participate in abscisic acid (ABA)-mediated gene expression [16]. AP2 family members play significant roles in the regulation of plant developmental processes, such as flower development [17], leaf development [18], bean (*Phaseolus vulgaris*)–*Rhizobium etli* symbiosis [19], and embryo development [20]. RAV family members have been characterized as regulators of plant abiotic and biotic responses [21] and plant development [22]. The Soloist is a unique member of the AP2/ERF superfamily, and it have been shown to regulate salicylic acid (SA) accumulation and are involved in defense against bacterial pathogens in *Arabidopsis thaliana* [23]. In addition, AP2/ERF transcription factors are considered as important targets for gene editing [24], which will help in studying the functions of the AP2/ERF superfamily. Together, these studies indicate that the identification and characterization of the AP2/ERF superfamily members are important for understanding the mechanisms of development and various responses in plants.

Recently, a number of draft genome sequences for both dicots and monocots have become available. Thus, genome-wide identification of the AP2/ERF superfamily members have been conducted in many plants, such as *Arabidopsis thaliana* [1], soybean (*Glycine max*) [25], barley (*Hordeum vulgare*) [26], grape (*Vitis vinifera*) [27], poplar (*Populus trichocarpa*) [28], Chinese cabbage (*Brassica rapa* ssp. *pekinensis*) [29], peach (*Prunus persica*) [30], and cucumber (*Cucumis sativus*) [31]. Comprehensive analyses have shown that the AP2/ERF superfamily plays important roles in plant development.

Longan (*Dimocarpus longan*), an important tropical/subtropical evergreen fruit tree, is cultivated widely in Southeast Asia, especially in China. Most longan tissues contain abundant bioactive compounds, including phenolic acids, flavonoids, and polysaccharides, that are of rich nutritional and medicinal value [32]. Longan embryo development has an extremely important role in fruit development, but it is a complicated process that is influenced by various factors, such as cell division and differentiation. Until now, no systematic analysis of the AP2/ERF superfamily has been conducted in longan, so the roles of members of this superfamily in somatic embryogenesis (SE) are still unclear. The longan genome was sequenced in our laboratory, which provides a great opportunity for the genome-wide study of the AP2/ERF superfamily [33]. In this study, we systematically and comprehensively characterized the AP2/ERF superfamily in longan using a number of different methods. We identified 125 longan AP2/ERF superfamily members that were divided into four families and classified into 13 groups. The gene structure, motif composition, phylogeny, *cis*-acting element, single nucleotide polymorphisms (SNPs), insertions and deletions (InDels), and alternative splicing (AS) events were analyzed and further investigated. Expression analysis was performed to identify the involvement of AP2/ERF members in non-embryogenic callus (NEC) and in embryogenic cultures, including embryogenic callus (EC), incomplete compact proembryogenic cultures (IcpEC), and globular embryos (GE) of the ‘Hong He Zi’ (HHZ) cultivar, as well as under different light quality and hormone treatments in EC and in nine organs of the ‘SI JI MI’ (SJM) cultivar. This study provides valuable clues for functional characterization of the AP2/ERF superfamily in longan.

## Results

### Identification of the AP2/ERF superfamily in longan

All candidate *AP2/ERF* genes corresponding to the Pfam AP2 domain (PF00847) were extracted from the longan genome dataset [33]. We annotated 125 distinct *AP2/ERF* putative genes based on the complete AP2 domains (Additional file 1: Table S1). A total of 101 genes with a single AP2 domain were assigned to the ERF family, which was divided into the ERF and DREB subfamilies with 61 and 40 genes respectively. A total of 19 genes were assigned to the AP2 family, and 13 of them contained two AP2 domains. The remaining six genes, Dlo_000585.2 (*AIL*, Additional file 1: Table. S1), Dlo_023666.1 (*WRI1*), Dlo_008555.1 (*ADAP*), Dlo_009234.2 (*RAP2.7*), Dlo_007774.3 (*RAP2.7*), and Dlo_012821.1 (*RAP2.7*), contained only one AP2 domain, but had high similarity with the AP2 family members and were distinct from the ERF family members. Because of this phylogenetic relationship, we assigned them to the AP2 family, which is consistent with the corresponding genes in *Arabidopsis* [1]. Four genes, which contained a single AP2 domain and a B3 domain, were assigned to the RAV family. The Dlo_030750.1, which was alone on a branch and close to the RAV family, was homologous to *Arabidopsis* At4g13040 (Soloist). Therefore, we found that the longan AP2/ERF (DlAP2/ERF) superfamily contained 101 ERF, 19 AP2, four RAV members, and one Soloist (Dlo_030750.1).

The following gene characteristics were analyzed: lengths of the coding sequence (CDS), protein sequence, protein isoelectric point (pI) and molecular weight (Additional file 1: Table S1). Among the 125 *DlAP2/ERF* genes, the complete CDS of one gene was not found (dlo_039227.1, *ERF-77*); the other 124 genes were analyzed further. Dlo_013715.1 (ERF-50) was the smallest protein with 123 amino acids (aa) and the largest protein was Dlo_011527.1 (Baby Boom, BBM) with 762 aa. The molecular weights of the proteins ranged from 13.1 to 83.3 kDa, and the Isoelectric point (pI) ranged from 4.53 (Dlo_001352.1, ERF-35) to 10.82 (Dlo_013715.1, ERF-50).

### Phylogenetic analysis and classification of the longan AP2/ERF superfamily

**Fig. 1:**
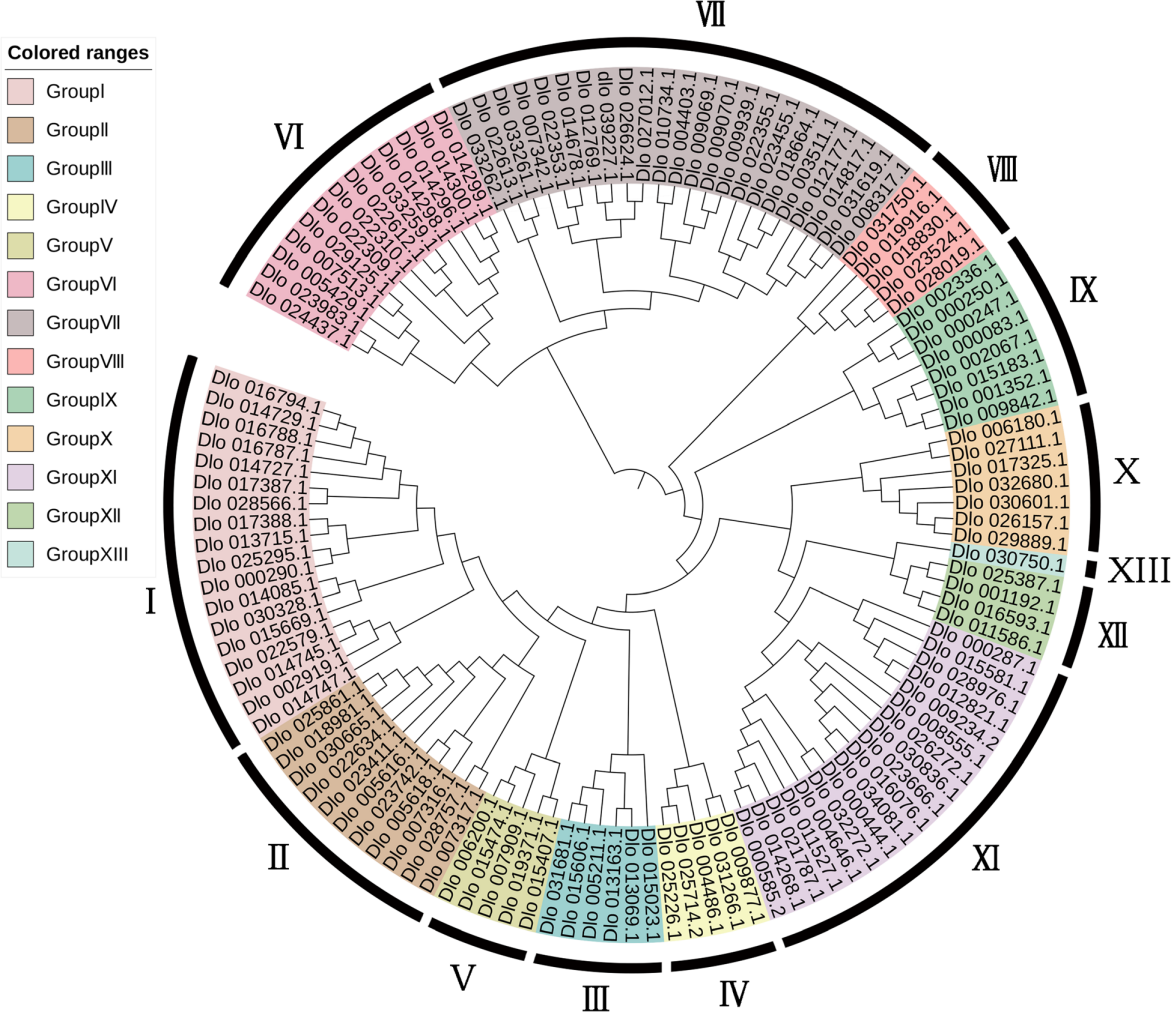
Phylogenetic tree used NJ method representing relationships among AP2/ERF superfamily of longan. The different-colored areas indicate different groups of AP2/ERF superfamily

The evolutionary relationship of the DlAP2/ERF superfamily was assessed by phylogenetic reconstruction using the conserved AP2 domain of the AP2/ERF proteins with the neighbor-joining (NJ) method (Fig. 1). The phylogenetic analysis indicated that the AP2/ERF superfamily divided into 13 groups, which is consistent with previous studies [1, 29]. Groups I–IV belonged to the DREB subfamily, groups V–X belonged to the ERF subfamily, and groups XI, XII, and XIII belonged to the AP2, RAV, and Soloist families, respectively. The Soloist gene (Dlo_030750.1) contained a single AP2 domain but was clustered with the RAV family, which is consistent with what was found in Chinese cabbage [29]. However, in grape, the Soloist gene was clustered with the AP2 family [27]. The in-depth phylogenetic analysis of the AP2 family members in group XI showed that they clustered into three subgroups, AP2-R1, AP2-R2, and AP2-R3 (Additional file 2: Figure S1).

**Fig. 2:**
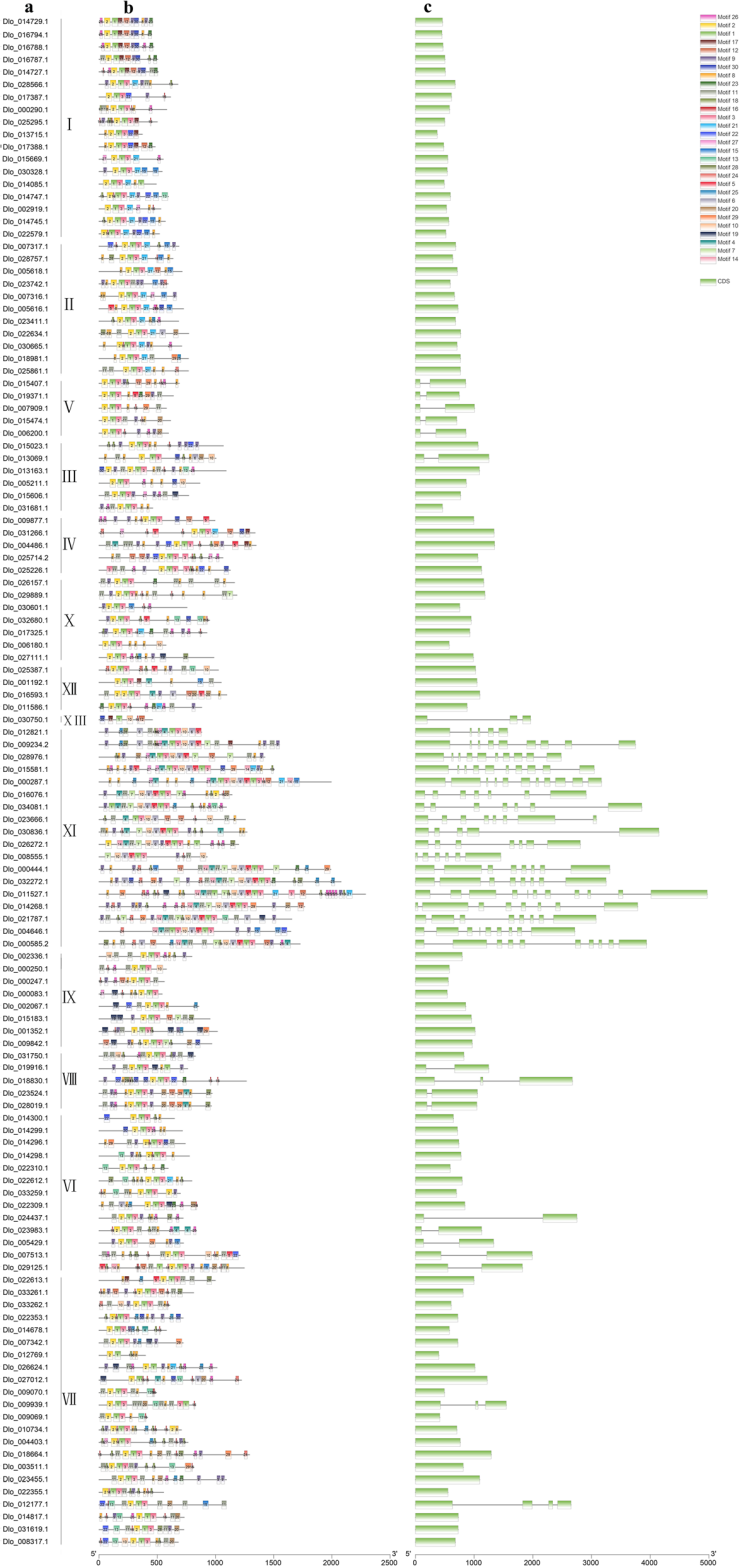
Architecture of conserved protein motifs and gene structure in the AP2/ERF superfamily of longan. **a** Gnen ID list of the DlAP2/ERF superfamily. **b** The motif composition of DlAP2/ERF proteins. The motifs, numbers 1–30, are displayed in different colored boxes. The sequence information for each motif is provided in Table S11. The length of protein can be estimated using the scale at the bottom. **c** Exon-intron structure of *DlAP2/ERF* genes. Green boxes indicate exons; black lines indicate introns

To further confirm the evolutionary relationship of the DlAP2/ERF superfamily, we also used a maximum likelihood (ML) method to assess the phylogenetic reconstruction (Additional file 3: Figure S2). The phylogenetic analysis indicated that the AP2/ERF superfamily clustered into four subfamilies, ERF, AP2, RAV, and Soloist, which is similar to the results obtained with the NJ method. The exceptions were Dlo_002336.1 (ERF-91), Dlo_000247.1 (ERF-2), and Dlo_000250.1 (ERF-3), which contain a single AP2 domain and were assigned to the ERF family but clustered with the DREB family with the ML method. The motif analysis showed that in Dlo_002336.1 (ERF-91), Dlo_000247.1 (ERF-2) and Dlo_000250.1 (ERF-3), the highly conserved AP2 domain was similar to that of the DREB family (Fig. 2b), so the different phylogenetic results between the NJ and ML method may be explained by the high similarity of the AP2 conserved domains.

### Motif composition and gene structure of the longan AP2/ERF superfamily

The conserved motifs and exon–intron organization of all identified *DlAP2/ERF* genes were examined to gain insights into the evolution of the DlAP2/ERF superfamily. The results showed that the different families among the AP2/ERF superfamily usually had different structures. For the conserved motif analysis among the DlAP2/ERF proteins, we used the MEME Suite of tools [34]. We identified a total of 30 motifs and used them to analyze the characteristics of the DlAP2/ERF superfamily proteins (Fig. 2b). The results showed that motif-1 was present in almost all AP2/ERF superfamily members, motif-2 was present in all ERF family members. Almost all ERF family members contained motif-1, motif-2, motif-3, and motif-4, and almost all AP2 family members contained motif-1, motif-3, motif-4, motif-9, and motif-14. Additionally, AP2/ERF superfamily members in the same groups usually shared a similar motif composition. For example, motif-5 and motif-6 were unique to the AP2 family, motif-13 and motif-17 were unique to the RAV family, and motif-23 was unique to the AP2-R3 subfamily.

Gene structure analysis showed that the ERF family members had from zero to three introns (Fig. 2c); 14 had one intron, two had two introns, one had three introns, and the remaining 83 members had no intron. All AP2 members and the Soloist gene had one to ten introns, whereas the RAV family had no intron. Further, genes within the same group usually had similar structures. For example, all DREB subfamily members had no intron, and all group V members, five group VI members also had no intron. The similarity of conserved motif composition and gene structure in the same subfamily indicated that the phylogenetic classification was reliable.

### *Cis*-acting elements in longan *AP2/ERF* genes

**Fig. 3:**
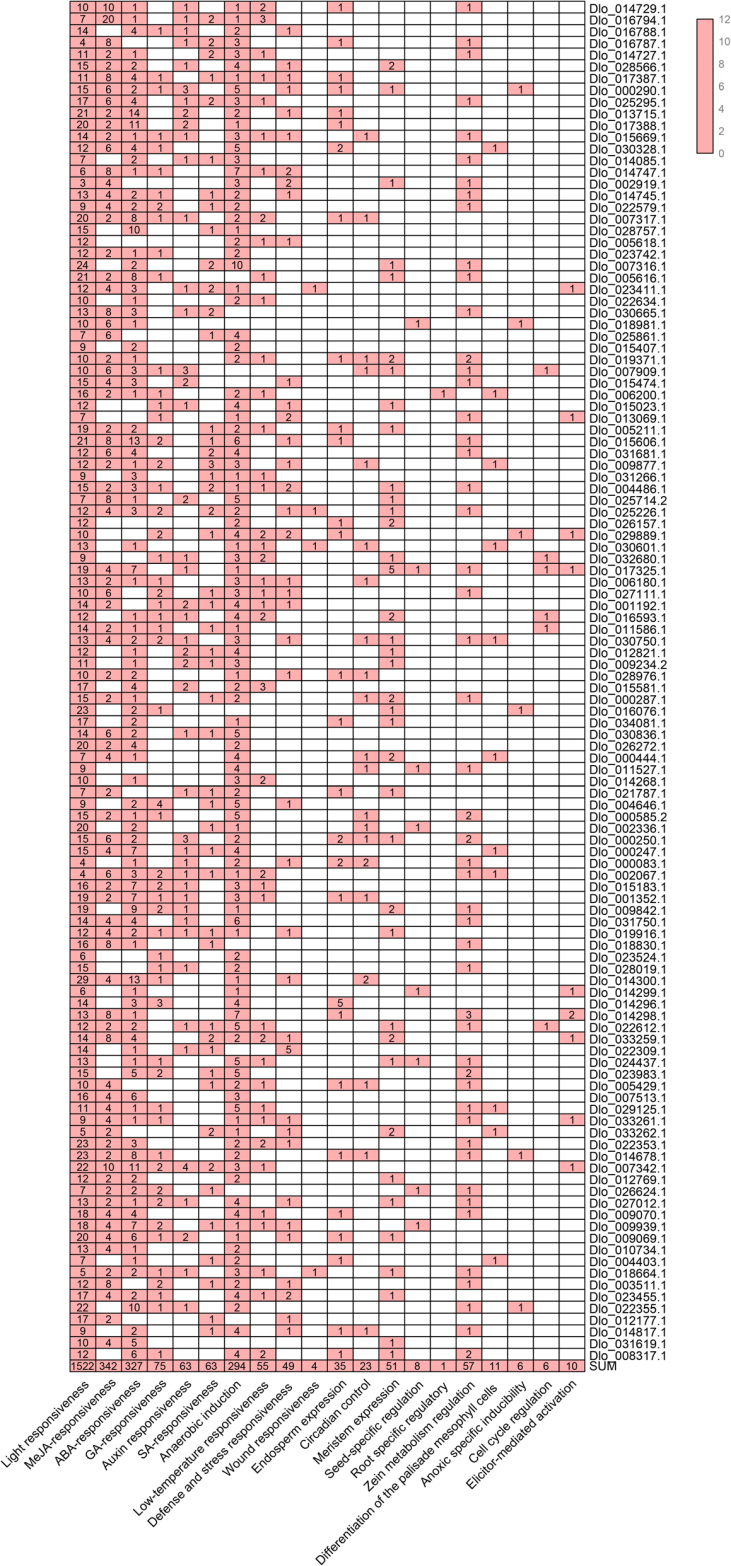
Predicted *cis*-acting elements in the promoter of the DlAP2/ERF superfamily. The 2.0 kb regulatory region upstream of ATG were analyzed with the PlantCARE software

To further analyze the potential function of *DlAP2/ERF* genes, a 2000-bp regulatory region upstream of the ATG (promoter) was searched for *cis*-acting elements analysis. Light-responsive (1522), hormone-responsive (870), and stress-responsive (402) boxes were detected in the promoter regions of *AP2/ERF* superfamily genes (Fig. 3; Additional file 4: Table S2). Hormone-related elements, such as methyl jasmonate (MeJA) (342), ABA (327), gibberellin (GA) (75), auxin (63), and SA (63) boxes, were often found in the promoter of *AP2/ERF* superfamily genes. Stress-related elements, such as anaerobic induction (294), low-temperature (55), defense (49), and wound induction (4) boxes, also were found in the promoter regions of *AP2/ERF* genes. Endosperm-related (35), circadian-related (23), meristem regulation (51), and seed regulation (8) *cis*-acting elements also were found in the AP2/ERF superfamily. These results indicated that *DlAP2/ERF* genes may be regulated by various *cis*-acting elements in their promoters during growth and stress responsive.

### RNA sequencing (RNA-seq) revealed SNPs and InDels in longan *AP2/ERF* genes in ‘HHZ’ and ‘SJM’ cultivars

**Fig. 4:**
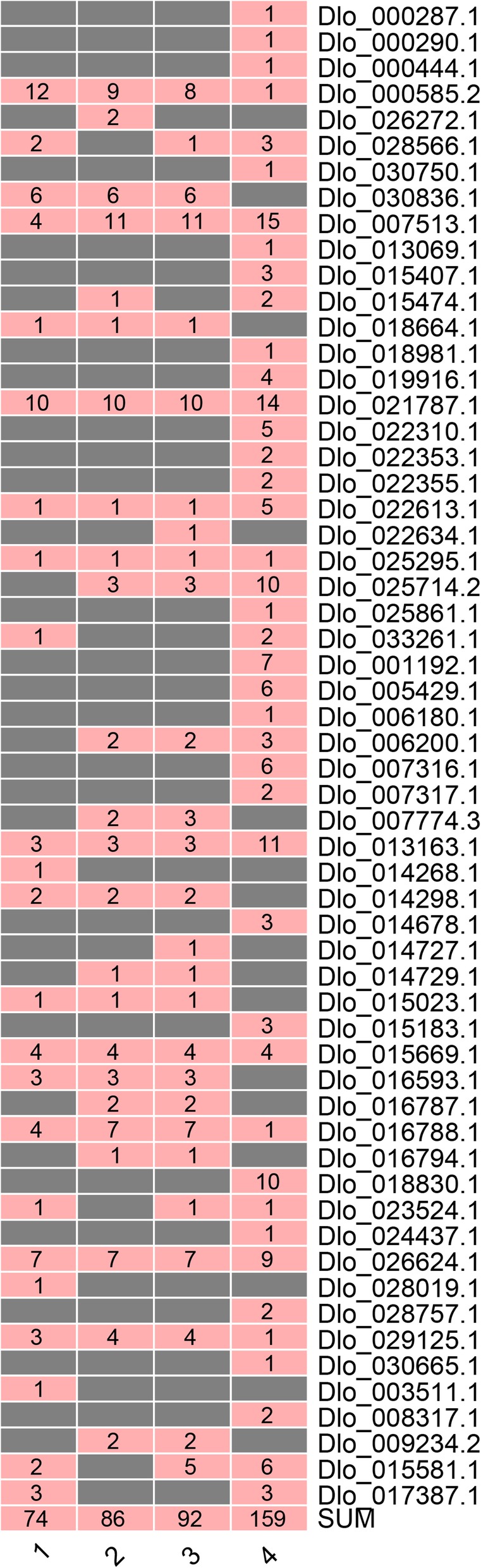
The SNP numbers of AP2/ERF superfamily detected in ‘HHZ’ and ‘SJM’ clutivars. **1** The SNP numbers of the AP2/ERF superfamily detected in the NEC and embryogenic cultures of ‘HHZ’ cultivar. **2** The SNP numbers of the AP2/ERF superfamily detected in the EC under different light quality treatments. **3** The SNP numbers of the AP2/ERF superfamily detected in the EC under different hormone treatments. **4** The SNP numbers of the AP2/ERF superfamily detected in nine organs of ‘SJM’ clutivar

**Fig. 5:**
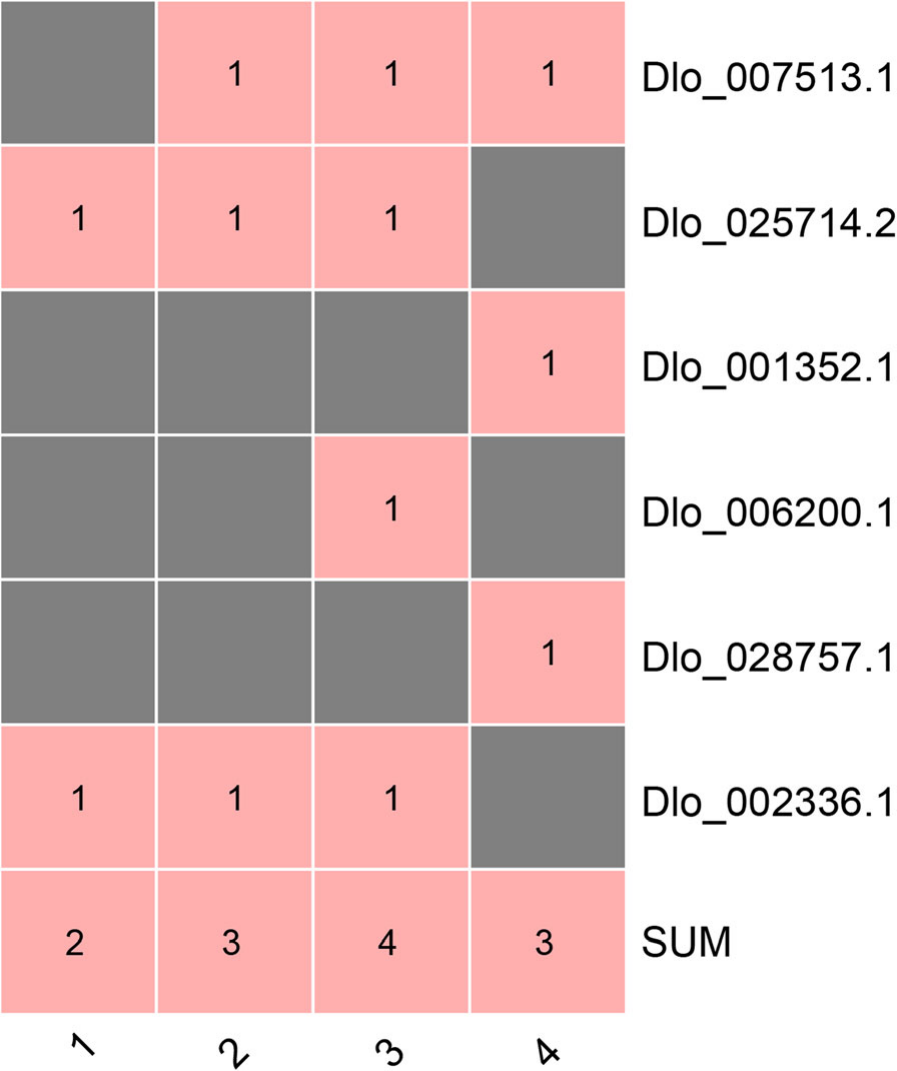
The InDel numbers of the AP2/ERF superfamily detected in ‘HHZ’ and ‘SJM’ clutivars. **1** The InDel numbers of the AP2/ERF superfamily detected in the NEC and embryogenic cultures of ‘HHZ’ cultivar. **2** The InDel numbers of the AP2/ERF superfamily detected in the EC under different light quality treatments. **3** The InDel numbers of the AP2/ERF superfamily detected in the EC under different hormone treatments. **4** The InDel numbers of the AP2/ERF superfamily detected in nine organs of ‘SJM’ clutivar

To investigate the variation of *DlAP2/ERF* superfamily genes, we extracted the SNP and InDel data from four transcriptome datasets corresponding to NEC and embryogenic cultures of the ‘HHZ’ cultivar, different light quality and hormone treatments in EC of the ‘HHZ’ cultivar, and nine organs of the ‘SJM’ cultivar (Figs. 4 and *5*; Additional file 5: Table S3 and Additional file 6: Table S4). We identified 74, 86, 92, and 159 SNPs, and 2, 3, 4, and 3 InDels in the *AP2/ERF* superfamily genes in these four datasets, respectively. Among then, Dlo_000585.1 (*AIL*), Dlo_007513.1 (*ERF-11*), Dlo_021787.1 (*AIL*), Dlo_013163.1 (*ERF-49*), and Dlo_026624.1 (*ERF-78*) had the highest number of SNPs in the ‘HHZ’ and ‘SJM’ cultivars; Dlo_000585.1 (*AIL*) had more SNPs in the ‘HHZ’ cultivar than in the ‘SJM’ cultivar, and Dlo_013163.1 (*ERF-49*) had more SNPs in the ‘SJM’ cultivar than in the ‘HHZ’ cultivar. In addition, 22 genes had SNPs that were detected only in the ‘SJM’ cultivar and five genes had SNPs that were detected in only the ‘HHZ’ cultivar. The different SNPs of the AP2/ERF superfamily members indicate the genetic diversity and could be used to develop variety-specific genetic markers.

### RNA-seq revealed alternative splicing (AS) events of longan *AP2/ERF* genes in different tissues and treatments

**Fig. 6:**
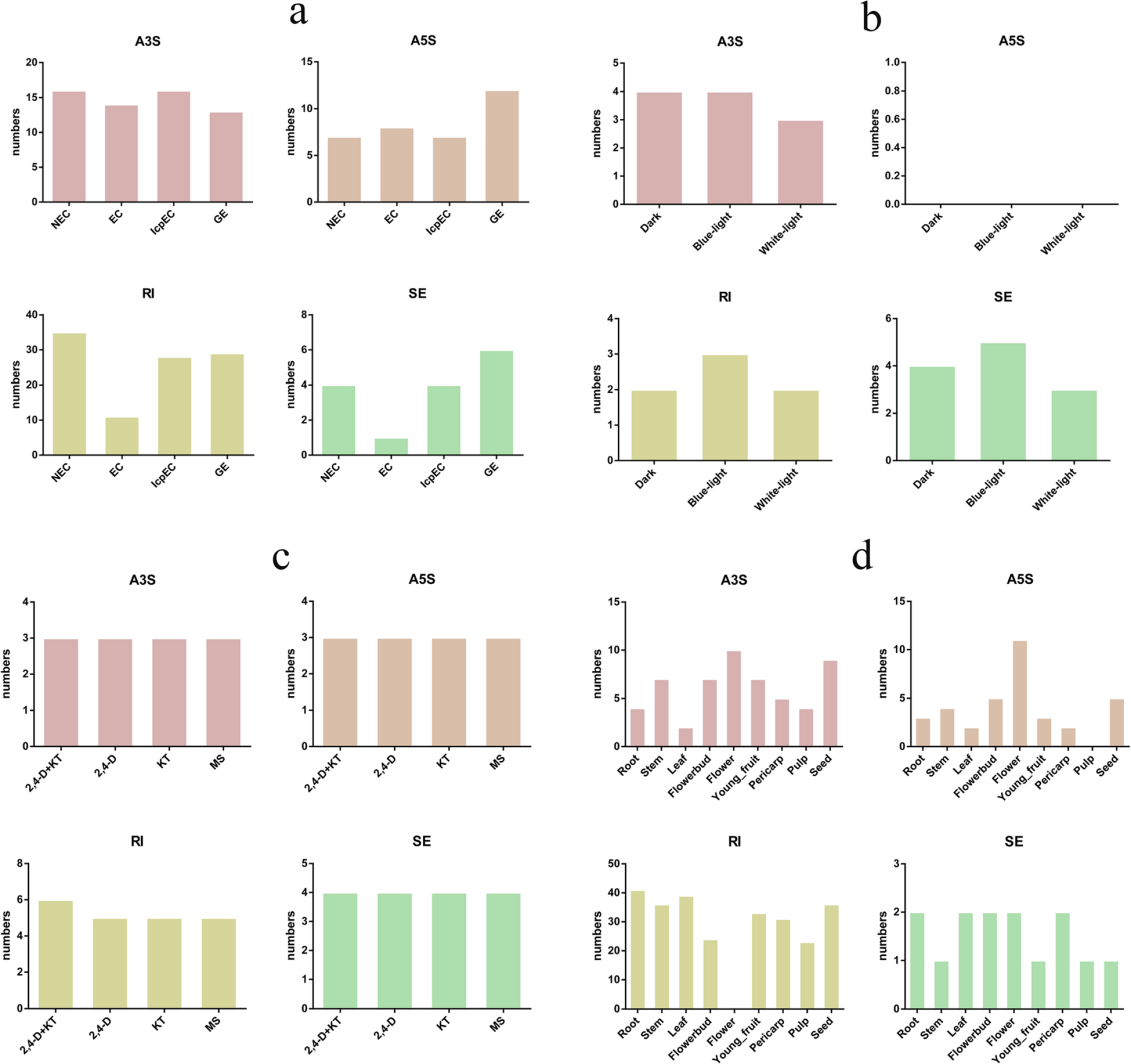
The AS events of the AP2/ERF superfamily detected in different tissues and in response to different treatments. **a** The AS events of the AP2/ERF superfamily detected in the NEC and embryogenic cultures of ‘HHZ’ cultivar. **b** The AS events of the AP2/ERF superfamily detected in the EC under different light quality treatments. **c** The AS events of the AP2/ERF superfamily detected in the EC under different hormone treatments. 2,4-D + KT, 2,4-D (1.0 mg/L) and KT (0.5 mg/L) were added to the MS medium; 2,4-D, only 2,4-D (1.0 mg/L) was added to the MS medium; KT, only KT (0.5 mg/L) was added to the MS medium; and MS, no hormone was added to the MS medium. **d** The AS events of the AP2/ERF superfamily detected in nine organs of ‘SJM’ clutivar. A3S represents alternative 3′ splice site acceptor, A5S represents alternative 5′ splice site donor, RI represents intron retention, SE and exon skipping

**Fig. 7:**
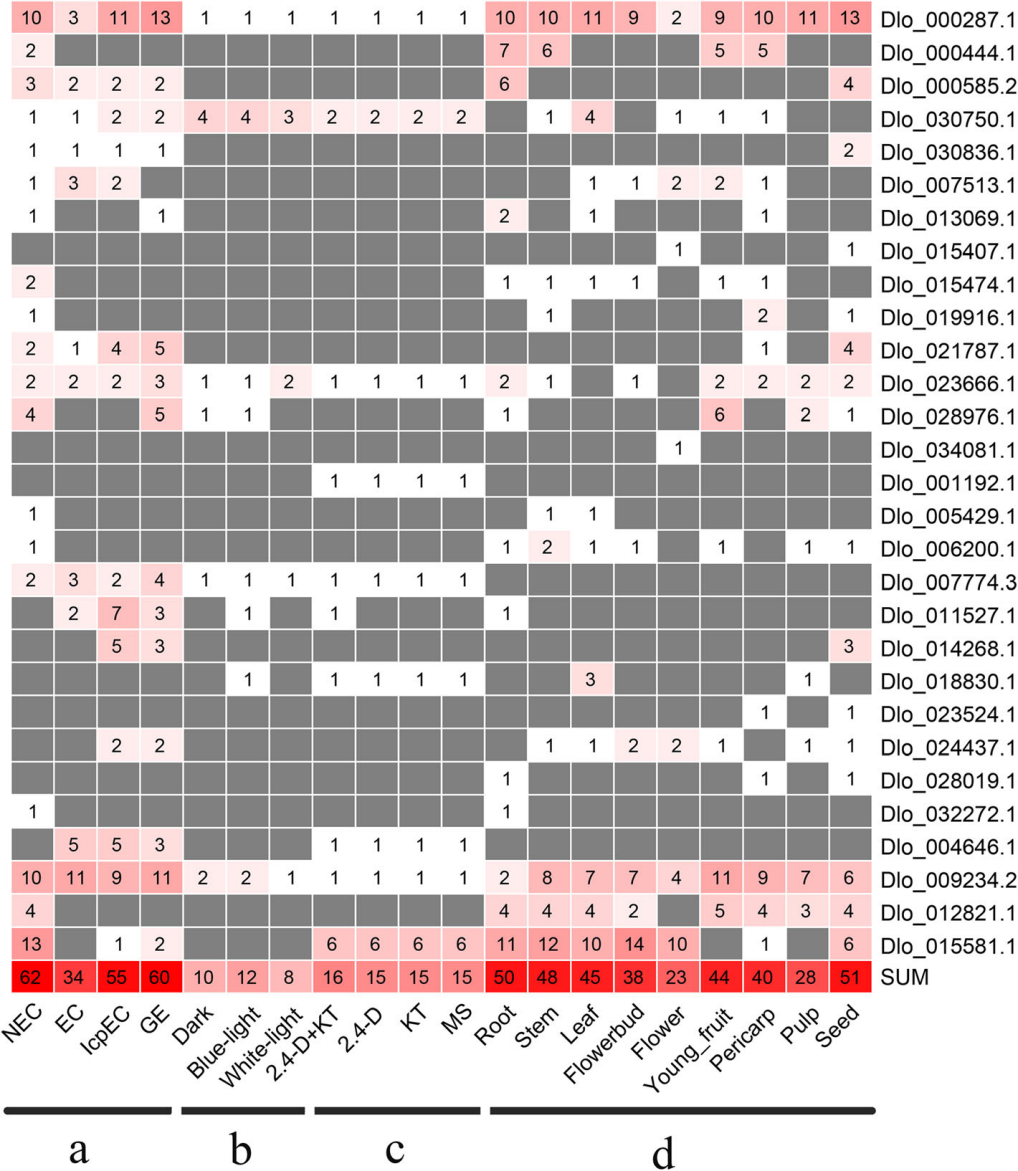
The AS events of the *DlAP2/ERF* genes detected in different tissues and in response to different treatments. **a** The AS events of the AP2/ERF superfamily detected in the NEC and embryogenic cultures of ‘HHZ’ cultivar. **b** The AS events of the AP2/ERF superfamily detected in the EC under different light quality treatments. **c** The AS events of the AP2/ERF superfamily detected in the EC under different hormone treatments. X-axis labels are the same as Fig. 6. **d** The AS events of the AP2/ERF superfamily detected in nine organs of ‘SJM’ clutivar

AS events regulate gene expression and expand proteomic diversity in plant development processes, and are regulated by the developmental stages and stress conditions. To investigate transcript expression and protein translation of AP2/ERF superfamily members, we examined four types of AS events across the NEC and embryogenic cultures (EC, IcpEC, and GE) of the ‘HHZ’ cultivar, different light quality and hormone treatments in EC of the ‘HHZ’ cultivar, and nine organs of the ‘SJM’ cultivar, including intron retention, exon skipping, alternative 5′ splice site donor, and alternative 3′ splice site acceptor (Fig. 6). The largest number of AS events in the AP2/ERF superfamily was detected in the NEC (62) stage, and the smallest number of AS events was found in the EC (34) stage (Fig. 7; Additional file 7: Table S5). The most and least types of AS events among these four stage were intron retention (103) and exon skipping (15), respectively. Nineteen of the 125 *DlAP2/ERF* genes had AS events in the NEC stage, followed by 15 genes in the GE stage, 14 in the IcpEC stage, and 11 in the EC stage (Fig. 7). Additionally, seven genes (Dlo_012821.1 (*RAP2.7*), Dlo_032272.1 (*ANT*), Dlo_006200.1 (*ERF-40*), Dlo_005429.1 (*ERF-38*), Dlo_019916.1 (*ERF-18*), Dlo_015474.1 (*ERF-14*), and Dlo_000444.1 (*ANT*)) had a total of 12 specific AS events in the NEC stage. Interestingly, no specific AS events were found in the EC, IcpEC, and GE stages. These results showed that the large number of specific AS events in the NEC stage may form various proteins with different functions, which may be important for the maintenance of NEC.

To confirm whether the AS events of AP2/ERF superfamily were affected by different light qualities and hormones, we detected four types of AS events from the transcriptome datasets (Fig. 7 and Additional file 8: Table S6). The highest number of AS events was detected in the blue-light treatment (12), followed by the dark (10) and white-light (8) treatments. Additionally, a total of eight genes had AS events in the blue-light treatment, followed by seven in the dark treatment and five in the white-light treatment (Fig. 7). Among the hormone treatments (2,4-dichlorophenoxyacetic acid (2,4-D) and kinetin (KT)), the highest number of AS events was detected in the 2,4-D + KT (16) treatment, followed by the 2,4-D (15), KT (15) and MS (Murashige and Skoog) (15) treatments. Further, 10 genes were found to undergo AS events in the 2,4-D + KT treatment, followed by nine genes in the 2,4-D, KT, and MS treatments (Fig. 7 and Additional file 9: Table S7). Additionally, Dlo_011527.1 (*BBM*) underwent specific AS event in the 2,4-D + KT treatment, and Dlo_018830.1 (*ERF-70*) and Dlo_011527.1 (*BBM*) underwent specific AS events in blue-light treatment. No specific AS events were detected in the dark, white-light, 2,4-D, KT, or MS treatments. We presumed that the AS events of Dlo_011527.1 (*BBM*) and Dlo_018830.1 (*ERF-70*) were affected by KT and blue-light quality.

By analyzing the ‘SJM’ cultivar transcriptome dataset, we found that the largest number of AS events in the AP2/ERF superfamily was in seed (51) and the smallest number was found in flower (23) (Additional file 10: Table S8). Among the types of AS events found in all nine organs, intron retention (263) was the most common and exon skipping (14) was the least common. A total of 16 *AP2/ERF* superfamily genes underwent AS events in seed, followed by 14 genes in root; the lowest number of AS events was found in flower (8) and pulp (8) (Fig. 7). Additionally, we detected five specific AS events in seed (Dlo_030836.1 (*WRI*) and Dlo_014268.1 (*ANT*)), and one specific AS event in root (Dlo_032272.1 (*ANT*) and flower (Dlo_034081.1 (*ADAP*), respectively. These results indicated that AS events can form diverse transcripts and may affect seed development. Overall, AS events are enriched in the NEC and seed stage, and they may also be affected by blue-light and KT.

### Expression profiling of longan *AP2/ERF* genes by RNA-seq

**Fig. 8:**
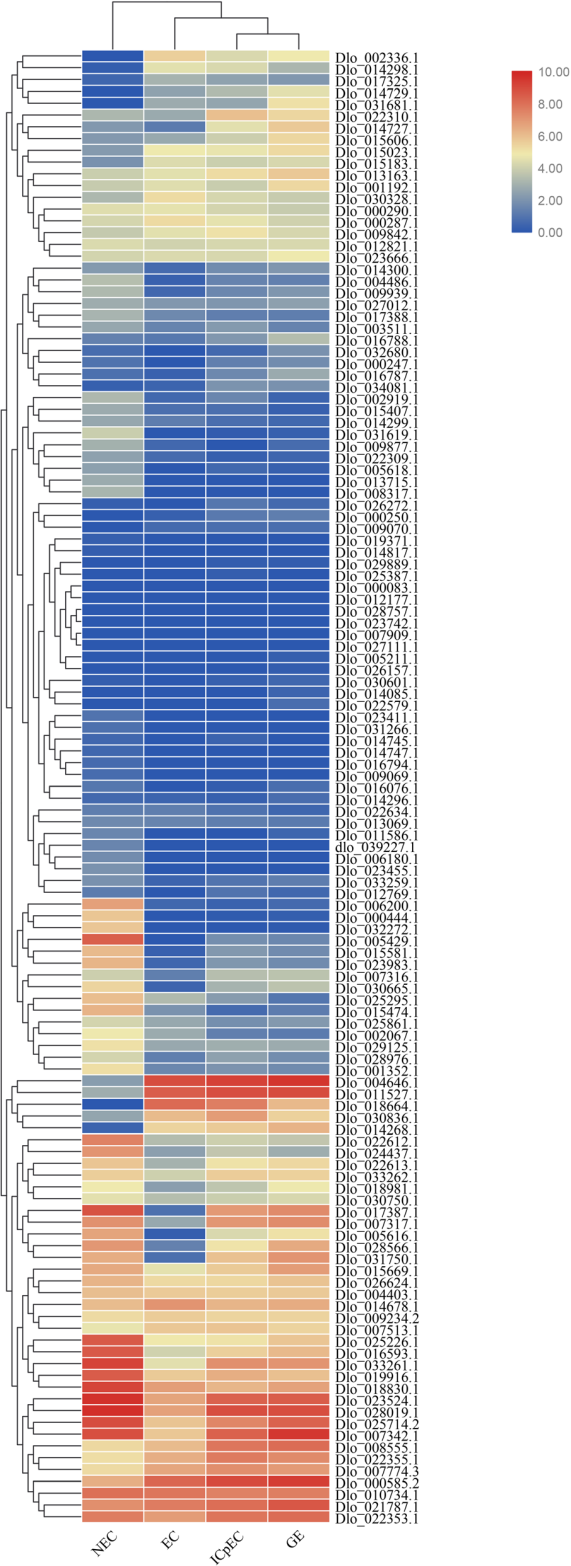
Hierachical clustering of expression profiles of longan *AP2/ERF* genes in the non-embryogenic callus and embryogenic cultures

To investigate whether the *AP2/ERF* superfamily genes affected early SE, the development of different organs, and respond to light and hormone in longan, we calculated the transcript levels in longan transcriptome datasets using FPKM values and constructed functional pathways. The four transcriptome datasets included the NEC and embryogenic cultures (EC, IcpEC, and GE) of the ‘HHZ’ cultivar, the ECs of ‘HHZ’ under different light quality and hormone treatments, and nine organs of the ‘SJM’ cultivar. We first analyzed the transcriptional levels of the *AP2/ERF* genes in the NEC and embryogenic cultures of the ‘HHZ’ cultivar (Fig. 8, Additional file 11: Table S9). Among the 125 *DlAP2/ERF* genes, six (Dlo_028757.1, Dlo_023742.1, Dlo_007909.1, Dlo_027111.1, Dlo_000083.1 and Dlo_012177.1) were not expressed in any of the tested samples; the remaining 119 genes were most abundant in the NEC stage (8897.38), followed by the GE (7626.92), IcpEC (6283.83), and EC (3746.59) stages. Among the 119 genes, 67, 31, 11, and 9 genes were most highly expressed in the NEC, GE, IcpEC, and EC stages, respectively. We found 78 differentially expressed *AP2/ERF* genes in NEC vs. EC, 41 in EC vs. IcpEC, and 18 in IcpEC vs. GE. Besides, Dlo_000444.1 (*ANT*), Dlo_032272.1 (*ANT*), Dlo_028976.1 (*RAP2.7*), and Dlo_015581.1 (*RAP2.7*) in the AP2 family, and Dlo_005429.1 (*ERF-38*), Dlo_001352.1 (*ERF-35*), Dlo_023983.1 (*ERF-75*), Dlo_015474.1 (*ERF-14*), Dlo_006200.1 (*ERF-40*), Dlo_025295.1 (*ERF-28*), Dlo_022612.1 (*ERF-23*), and Dlo_024437.1 (*ERF-76*) in the ERF family were specifically expressed in the NEC stage, with extremely low expression levels in the other stages. Thus, these genes can be used as marker genes in the NEC stage. We presumed that *DlAP2/ERF* genes may effect mainly the development of NEC and GE, and affect the transition from NEC to EC.

To analyse whether the DlAP2/ERF superfamily responds to light quality, we analyzed the transcript levels of the *DlAP2/ERF* genes in ECs with blue-light, white-light, and dark treatments using FPKM values from the transcriptome dataset. We found 106 expressed *AP2/ERF* genes in the transcriptome dataset (Additional file 12: Figure S3, Additional file 11: Table S9). They were most abundant in the dark treatment (6818.18), followed by white-light (3448.89) and blue-light (3071.69) treatments. A total of 34, 38, and 3 genes were differentially expressed in white-light vs. dark, dark vs. blue-light, and white-light vs. blue-light comparisons, respectively. Further, Dlo_004646.1 (*PLT*) and Dlo_000585.2 (*AIL*) had the highest expression levels in the white-light and blue-light treatments, respectively. These results showed that light may affect the expression of *DlAP2/ERF* genes but they may not be influenced by light quality changes.

To investigate whether the AP2/ERF superfamily responds to 2,4-D and KT, the expression levels of the *DlAP2/ERF* genes in the transcriptome dataset from hormone treatments (2,4-D and KT) were analyzed. Among the 125 *AP2/ERF* genes, 107 were detected in the transcriptome dataset; the other 18 were not detected (Additional file 13: Figure S4 and Additional file 11: Table S9). *AP2/ERF* genes were most abundant in the 2,4-D + KT (5770.59) and 2,4-D (5582.93) treatments. The MS (5149.25), and KT (4932.11) treatments, which lacked auxin (2,4-D) in the medium, had the lowest expression levels. Additionally, we detected 10 differentially expressed genes in 2,4-D + KT vs. MS, followed by 2,4-D vs. MS (8), 2,4-D + KT vs. KT (7), and 2,4-D vs. KT (5). Only one and zero differentially expressed genes were detected in 2,4-D + KT vs. 2,4-D and KT vs. MS. These results indicate that some *DlAP2/ERF* genes may be affected by 2,4-D.

We also analyzed the transcriptional levels of *DlAP2/ERF* genes in nine organs of the ‘SJM’ cultivar (Additional file 14: Figure S5, Additional file 11: Table S9). Among the 125 genes, we detected 120 genes that were expressed in all nine organs, three (Dlo_026157.1, Dlo_025387.1 and Dlo_012769.1) that were expressed in some of the organs, and two (Dlo_012177.1 and Dlo_008555.1) that were not detected in any of the organs. The *AP2/ERF* genes were most abundant in pericarp (14196), followed by stem (12807) and leaf (11765), and least abundant in root (3583) and seed (3831). Some of the genes were specifically expressed in different organs. For example, Dlo_015606.1 (*ERF-64*), Dlo_021787.1 (*AIL*), Dlo_014268.1 (*ANT*), and Dlo_000585.2 (*AIL*) were specifically expressed in seed, and Dlo_015581.1 (*RAP2.7*) was specifically expressed in flower bud and flower (Additional file 14: Figure S5). The results indicated that the AP2/ERF superfamily may widely regulates the developmental processes of longan because of its role in the development of NEC and embryogenic cultures and various tissues, and may be induced by 2,4-D and light.

### Expression patterns of *AP2/ERF* genes in NEC and embryogenic cultures of longan and the response to hormones by qRT-PCR

**Fig. 9:**
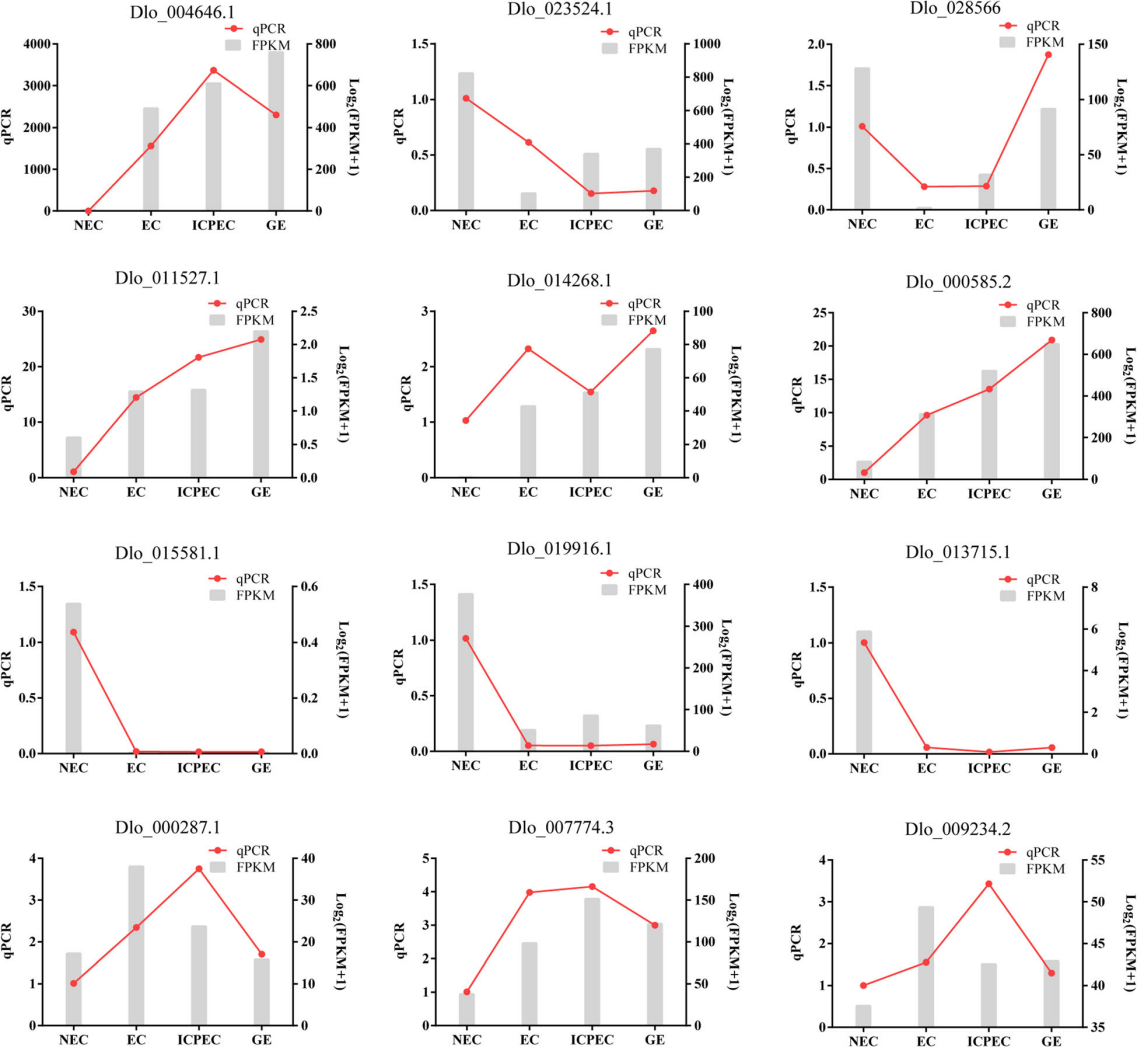
Expression analysis of 12 selected *DlAP2/ERF* genes in the non-embryogenic callus and embryogenic cultures of longan by qRT-PCR. Red line graph represents qRT-PCR results; Gray columnar section represents FPKM values

To confirm the transcriptional regulation of the *DlAP2/ERF* genes in the NEC and embryogenic cultures of the ‘HHZ’ cultivar, 12 differentially expressed *DlAP2/ERF* genes were selected for validation by qRT-PCR (Fig. 9). The qRT-PCR results showed that seven of the 12 selected genes had the same expression trends as those obtained by RNA-seq. Among them, Dlo_023524.1 (*ERF-73*) had the highest expression level in the NEC stage; Dlo_004646.1 (*PLT*), Dlo_000287.1 (*AP2*), Dlo_007774.3 (*RAP2.7*), and Dlo_009234.2 (*RAP2.7*) had the highest expression levels in the IcpEC stage; and Dlo_028566.1 (*ERF-6*), Dlo_011527.1 (*BBM*), Dlo_014268.1 (*ANT*)s and Dlo_000585.1 (*AIL*) had highest expression levels in the GE stage. Specially, the expression levels of Dlo_011527.1 (*BBM*) and Dlo_000585.1 (*AIL*) increased from the NEC to GE stage and reached their peaks in the GE stage. Interestingly, Dlo_015581.1 (*RAP2.7*), Dlo_019916.1 (*ERF-18*), and Dlo_013715.1 (*ERF-50*) were specifically expressed in the NEC stage, indicating they may marker genes for the NEC stage. Because of the high expression levels of *AP2* genes in the IcpEC and GE stage, we presumed that the *AP2* and *AIL* subfamily genes may promote the formation and development of GE.

**Fig. 10:**
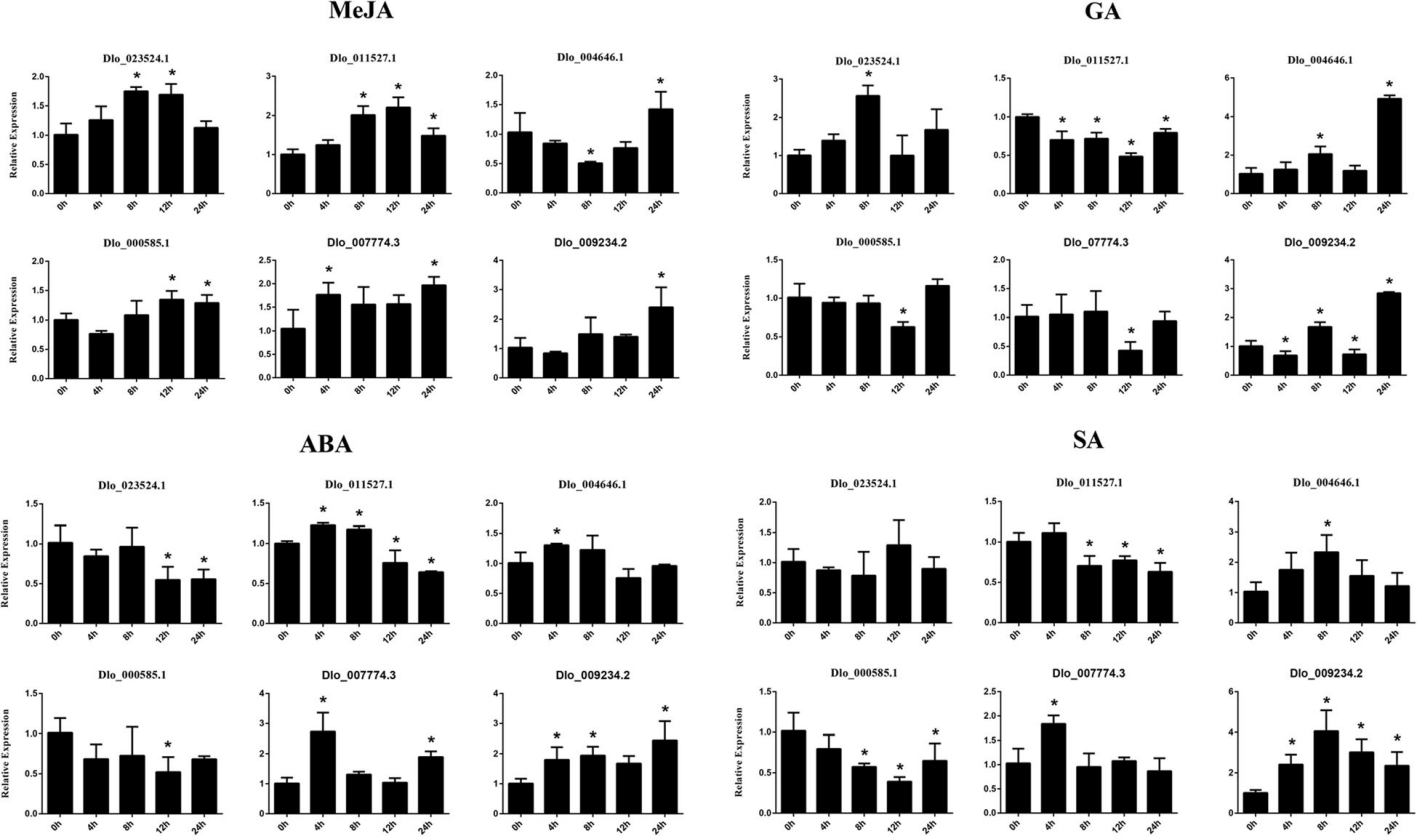
Expression analysis of six selected *DlAP2/ERF* genes respond to four hormone treatments by qRT-PCR. Data were normalized to *EF1α* and *ACTB* gene and vertical bars indicate standard deviation. Asterisks indicate the corresponding gene significantly up- or down-regulated compared with the untreated control (*P < 0.05)

According to the *cis*-acting elements in the AP2/ERF superfamily, we chose six of the 12 validated genes to confirm by qRT-PCR to confirm whether the expression patterns were influenced by different hormone treatments (SA, GA, ABA, and MeJA) (Fig. 10). We found that some of the *DlAP2/ERF* genes were significantly induced by multiple hormone treatments, and others were repressed. For instance, Dlo_023524.1 (*ERF-73*), Dlo_011527.1 (*BBM*), Dlo_000585.1 (*AIL*), Dlo_007774.3 (*RAP2.7*), and Dlo_009234.2 (*RAP2.7*) were significantly induced by MeJA treatment, whereas Dlo_004646.1 (*PLT*) was down-regulated and then up-regulated by MeJA treatment. Dlo_023524.1 (*ERF-73*) and Dlo_004646.1 (*PLT*) were induced by GA treatment, Dlo_011527.1 (*BBM*), Dlo_000585.1 (*AIL*), and Dlo_007774.3 (*RAP2.7*) were repressed by GA treatment, and Dlo_009234.2 (*RAP2.7*) was induced then repressed by GA treatment at different times. Dlo_004646.1 (*PLT*), Dlo_007774.3 (*RAP2.7*), and Dlo_009234.2 (*RAP2.7*) were induced by ABA treatment, Dlo_023524.1 (*ERF-73*) and Dlo_000585.1 (*AIL*) were repressed by ABA treatment, and Dlo_011527.1 (*BBM*) was induced then repressed by ABA treatment. Dlo_004646.1 (*PLT*), Dlo_007774.3 (*RAP2.7*), and Dlo_009234.2 (*RAP2.7*) were induced by SA treatment, whereas Dlo_011527.1 (*BBM*) and Dlo_000585.1 (*AIL*) were repressed by SA treatment. Interestingly, three genes, Dlo_011527.1 (*BBM*), Dlo_004646.1 (*PLT*), and Dlo_000585.1 (*AIL*), showed opposite expression patterns under the MeJA and SA treatments. Overall, the candidate genes were influenced by different hormone in various ways.

### AP2/ERF protein interactions among specific proteins in longan

**Fig. 11:**
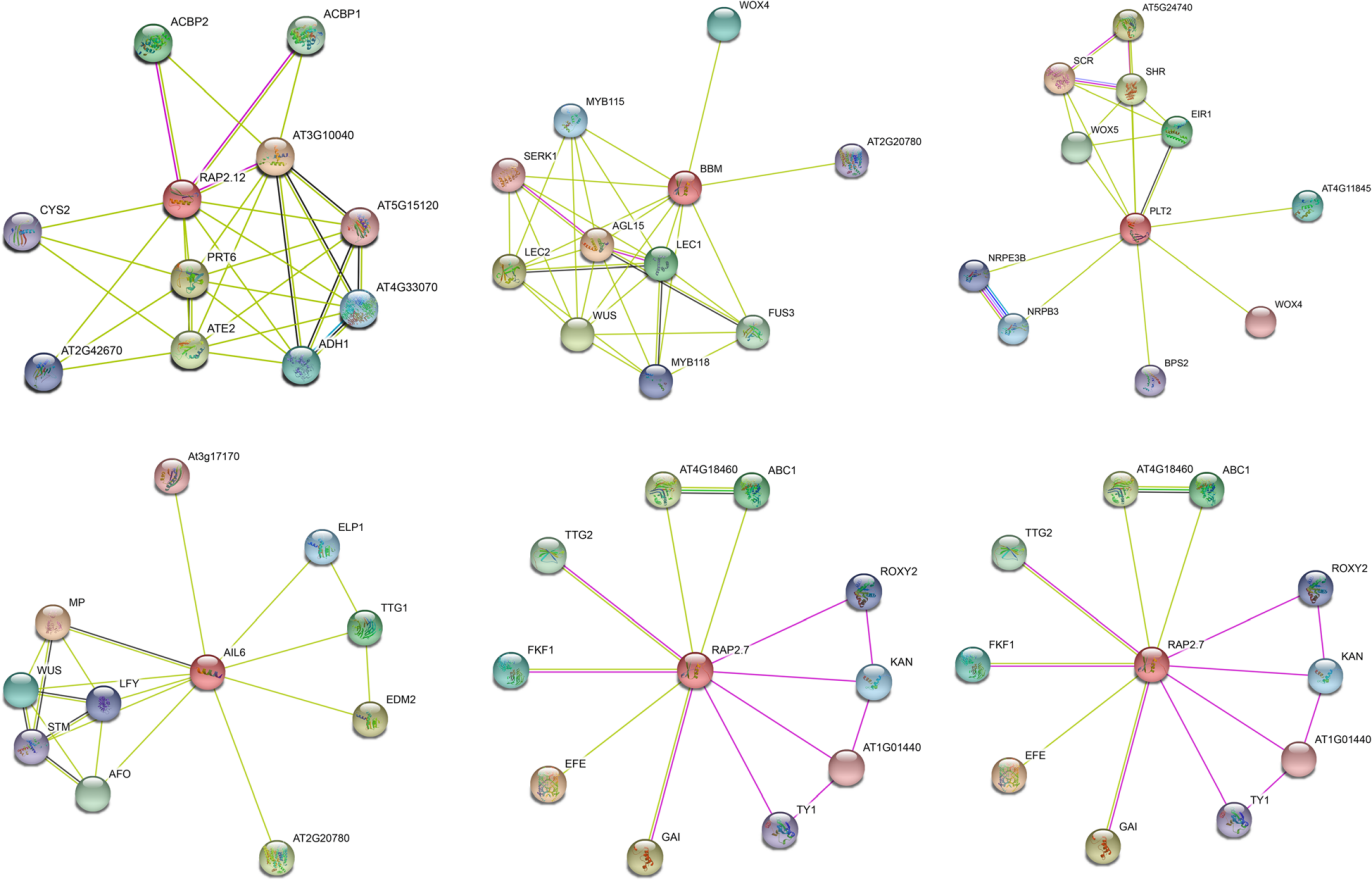
Interaction network of six selected DlAP2/ERF proteins. The results were based on an *Arabidopsis* association model

To confirm the potential functions of the DlAP2/ERF proteins, we selected six validated differentially expressed genes to construct a protein–protein interaction network using STRING 10.5 software based on an *Arabidopsis* association model (Fig. 11). Dlo_023524.1, which shared high homology with AtRAP2.12, interacts with ACBP1, ACBP2, CYS2, and PRT6. Dlo_011527.1, which shared homology with AtBBM, interacts with MYB115, SERK1, AGL15, LEC1, and FUS3. Dlo_004646.1,which shared homology with AtPLT2, interacts with WOX5, SHR, EIR1, WOX4, BPS2, NRPB3, and NRPE3B. Dlo_000585.1, which shared high homology with AtAIL6, interacts with ELP1, TTG1, EDM2 AFO, LFY, WUS, and MP proteins. Dlo_007774.3 and Dlo_009234.2, which shared homology with AtRAP2.7, interact with TGG2, FKF1, EFE, GAI, TY1, KAN, and ROXY2.

## Discussion

### The longan AP2/ERF superfamily is diverse in evolution process

The AP2/ERF superfamily comprises a large family of transcription factors that are ubiquitous to all plant species. Members of the AP2/ERF superfamily have been identified in many species whose genomes have been sequenced [1, 29, 35, 36]. In this study, we identified 125 members of the AP2/ERF superfamily in the longan genome, including 101 ERF, 19 AP2, four RAV family members, and one Soloist. The number of *AP2/ERF* genes in longan (125) is slightly lower than the numbers in *Arabidopsis* (148), cucumber (131), and *Vitis vinifera* (132), and much lower than that in Chinese cabbage (291) and poplar (202). The genomes of these plants have different sizes, namely 471 Mb in longan, 283 Mb in Chinese cabbage, and 475 Mb in *Vitis vinifera*, which shows that the number of AP2/ERF superfamily members is not absolutely correlated with genome size. However, we detected 101 ERF family members in longan compared with the 122 in *Arabidopsis* and 169 in poplar that have been identified, which indicates that the different numbers of the AP2/ERF superfamily members in these plants may be due to the different numbers of ERF family members.

Transcription factor motifs and domains are often related to transcriptional activity and protein interaction. The motif analysis of the DlAP2/ERF superfamily showed that most of the AP2/ERF superfamily members contained motif-1, motif-2, motif-3, and motif-4 related to the AP2 domain. This result is similar to what was found in tartary buckwheat [37]. Motif-6 and motif-10 were detected specifically in the AP2 family, indicating that they may play a unique role in the AP2 family. Although some motifs of the AP2/ERF superfamily were highly conserved, the unique motifs in different groups may have important functions in longan; however, these functions require further investigation. Structural analysis of the AP2/ERF superfamily showed that 82% of the *DlERF* and all of the *DlRAV* genes had no intron, whereas the *AP2* family and Soloist genes had from one to nine introns, which is similar to what was found for *AP2/ERF* genes in tartary buckwheat [37]. The differences between the structures of *ERF*, *RAV*, and *AP2* family genes indicated that they had largely differentiated during the evolution of the longan genome, and thus may have great functional differentiation.

### SNPs, InDels, and AS events exist in the longan AP2/ERF superfamily

Next-generation sequencing platforms have accelerated the discovery of SNPs and InDels, and the development of a large number of SNP and InDel markers. SNP and InDel markers are used to study genetic polymorphisms in cultivars, including breeding lines and mapping populations, and have been widely used in many species, such as maize [38], soybean [39], peach [40], and longan [41]. However, few studies have focused on SNPs and InDels in the AP2/ERF superfamily. In this study, we identified SNPs and InDels in *AP2/ERF* superfamily genes using longan genome and transcriptome datasets, including NEC and embryogenic cultures of the ‘HHZ’ cultivar, different light quality and hormone treatments in ECs of the ‘HHZ’ cultivar, and nine organs of the ‘SJM’ cultivar. We found that Dlo_007513.1 (*ERF-11*), Dlo_021787.1 (*AIL*), and Dlo_026624.1 (*ERF-78*) had a large number of SNPs in the ‘SJM’ cultivar dataset. Dlo_000585.2 (*AIL*) and Dlo_021787.1 (*AIL*), which had a large number of SNPs in ‘HHZ’ cultivar dataset, also had increased expression levels in early SE. Their AS events also were detected in early SE, which may promote the development of early SE in longan. Therefore, we predicted that the *AIL* genes involved in the development of SE in longan may have evolved rapidly.

The removal of introns from immature mRNAs by a “pre-mRNA splicing” process is known to occur in the vast majority of eukaryotic protein-coding genes, resulting in various transcripts being encoded by the same gene [42]. About 80% of the nuclear genes in plant genomes were reported to contain non-coding introns, and the dominant mechanisms in AS are intron retention, exon skipping, and alternative 5′ and 3′ splice sites [43–45]. We found that intron retention also was the main AS events in the DlAP2/ERF superfamily. AS events affect transcript export, protein translation, and degradation [46]. In plants, AS events can change during the growth and developmental processes, such as seed development, flowering time, and circadian clock, and under biotic and abiotic stresses [47]. Up to now, few reports on changes in AS events of the *AP2/ERF* superfamily genes are available. The direct evidence for AS events found in the current study revealed a new layer of complexity in the developmental regulation of *DlAP2/ERF* genes. Among the NEC and embryogenic cultures, Dlo_000287.1 (*AP2*), Dlo_015581.1 (*RAP2.7*), and Dlo_009234.2 (*RAP2.7*) showed relative high numbers of AS events in the NEC stage; Dlo_009234.2 (*RAP2.7*) showed high numbers in the EC stage; and Dlo_000287.1 (*AP2*) and Dlo_009234.2 (*RAP2.7*) showed high numbers in the IcpEC and GE stages. These three genes, which share homology with *AtAP2*, may regulate the embryogenesis of longan by having diverse AS transcripts. Besides, Dlo_000287.1 (*AP2*), Dlo_015581.1 (*RAP2.7*), and Dlo_009234.2 (*RAP2.7*) also showed high numbers of AS events in multiple organs of the ‘SJM’ cultivar. For example, Dlo_000287.1 (*AP2*) showed the highest numbers of AS events in seed, and Dlo_015581.1 (*RAP2.7*) showed the highest numbers in flower bud. An *AP2* gene in orchid was reported to have AS forms; namely, a long transcript *OitaAP2* and a short transcript *OitaAP2_ISO* in which the last exon is skipped [48]. AS variants also were identified in the *AP2* genes of kiwifruit, where the AS ninth exon flanked by two introns was followed by the tenth exon [49]. Thus, AS events form proteins with different functions and these proteins participate in the development of various organs in longan. The variations in SNPs, InDels, and AS events are likely to results in changes in gene expression levels and protein polymorphisms that are reflected in the phenotype, thereby contributing to the growth and environmental adaption of longan. All of these results require further validation.

### AP2/ERF members regulate early SE and organs development in longan, and respond to hormones such as MeJA, SA, ABA, and 2,4-D

There is considerable evidence that the AP2/ERF superfamily plays important roles in regulating plant growth and development, and in conferring tolerance to abiotic and biotic stresses [50–54]. Hormones not only play crucial roles in various plant growth processes, but are also involved in signaling and gene expression during environmental stresses. The functions of genes can be preliminarily predicted by analyzing gene expression patterns. Among the AP2/ERF supefamily Dlo_011527.1 (*BBM*) had the highest expression level in the GE stage. Dlo_011527.1 (*BBM*), Dlo_021787.1 (*AIL*), Dlo_014268.1 (*ANT*), Dlo_004646.1 (*PLT*), and Dlo_000585.2 (*AIL*), which clustered in the AIL branch in the AP2 family, were reported to promote SE in *Arabidopsis* and had extensive overlap functions, indicating they may have similar functions in longan [20]*.* Moreover, the BBM protein has been reported to interact with the LAFL/AGL15 network, which includes LEC1, LEC2, ABI3, FUS3, and AGL15, and may act upstream of the AIL branch. In *Arabidopsis*, BBM transcriptionally regulated *LAFL* genes, LEC2 and ABI3 quantitatively regulated BBM-mediated SE, and FUS3 and LEC1 were found to be essential for this process [20]. Therefore, it is necessary to verify whether the BBM (AIL)-LAFL/AGL15 network in longan functions in a similar as the *Arabidopsis* network and regulates SE in longan. The qRT-PCR results showed that Dlo_011527.1 (*BBM*), Dlo_004646.1 (*PLT*), and Dlo_000585.1 (*AIL*) had inverse expression patterns under MeJA and SA treatments, which may regulated the downstream genes to respond to hormone, and indicated that *AIL* genes may be induced by the MeJA and SA signaling pathway in longan. It has been reported that MeJA regulates the development of SE in *Medicago sativa* [55], and jasmonic acid (JA) and SA play antagonistic roles in *Arabidopsis* where SA strongly suppresses the JA-dependent pathway [56]. We found that SA and MeJA may also play antagonistic roles in regulating some *DlAP2/ERF* genes. Overall, the *AIL* branch may respond to MeJA and SA and participate in the development of early SE in longan.

Dlo_009234.2 (*RAP2.7*) is an *AP2* gene that showed high expression level in the IcpEC stage and may regulate early SE, especially the formation of GE. Besides, Dlo_009234.2 (*RAP2.7*), which has an ABA response element, was significantly induced by ABA treatment. ABA plays an important role in plant embryo development and promotes the synthesis and accumulation of nutrients such as proteins, lipids, and starch [57–60]. ABA promotes early SE of *Japanese larch*, which can affect the transformation of SE from proliferative division to maturation, and increases the dehydration tolerance of SEs and prevents premature germination to improve embryo quality [61]. Together these results suggest that Dlo_009234.2 (*RAP2.7*) may be induced by ABA and participate in the SE of longan.

The hormone 2,4-D is an important growth regulator that is used in embryogenic cell maintenance and that can inhibit somatic embryo initiation [62]. We found that more *DlAP2/ERF* genes were differentially expressed under 2,4-D treatment compared with KT treatment and had higher expression levels in the 2,4-D treatment group, which showed that some *AP2/ER*F genes may be induced intensively by 2,4-D treatment. Dlo_014727.1 (*ERF-57*) had the highest expression levels in 2,4-D + KT treatments compared with the other three treatments, suggesting that Dlo_014727.1 (*ERF-57*) may be induced by exogenous 2,4-D and participate in maintaining the embryogenic callus.

Tissue-specific expression profiles indicated that most of the *DlAP2/ERF* genes were expressed in almost all organs (96%), and 57.9% of the *AP2* family genes were expressed at high levels in root, which is similar to what was found in Chinese cabbage and tartary buckwheat [29, 37]. The phylogenetic analysis showed that two *AP2* genes, Dlo_009234.2 (*RAP2.7*) and Dlo_007774.3 (*RAP2.7*) that were highly expressed in young fruit, flower bud, and flower clustered together. Dlo_015581.1 (*RAP2.7*), which was highly expressed in flower bud and flower, clustered with Dlo_009234.2 (*RAP2.7*) and Dlo_007774.3 (*RAP2.7*). Subsequently, Dlo_015581.1 (*RAP2.7*), Dlo_009234.2 (*RAP2.7*) and Dlo_007774.3 (*RAP2.7*), which revealed they may have a similar evolutionary relationship, was found to share homology with *AtRAP2.7* (AT2G28550.1). Moreover, *RAP2.7* was identified as an *AP2* gene that promoted flower growth [63], thereby providing a basis to further verify the functionality of *AP2* genes.

Seed growth and developmental processes are particularly important for the survival of all seed plants. Interestingly, a DREB subfamily member, Dlo_015606.1 (*ERF-64*), which shares homology with *AtRAP2.1* (AT1G46768.1), was expressed specifically in longan seed. In *Arabidopsis*, RAP2.1 binds to the DRE/CRT domain and is activated by drought and cold stresses [64]. RAP2.1 also functions as an active transcriptional repressor similar to AtERF7. AtERF7 (RAP2.9), which shares homology with Dlo_017387.1 (ERF-100), binds specifically to the GCC-box and recruits the co-repressor AtSin3 and HDA19, a histone deacetylase. The transcriptional repression activity of AtERF7 was enhanced by HDA19 and AtSin3 [65]. Dlo_015606.1 (*ERF-64*) and Dlo_017387.1 (*ERF-100*) both belong to the DRBE subfamily and may also be active repressors in longan. Therefore, whether Dlo_015606.1 (*ERF-64*) and Dlo_017387.1 (*ERF-100*) have similar functions in regulating the development of longan needs to be investigated.

Overall, these findings provide insights into the potential functional roles of the AP2/ERF superfamily members. The comprehensive analyses will help in selecting candidate *DlAP2/ERF* genes for further functional research and provides new insights into longan embryo development.

## Conclusions

The comprehensive analysis of the longan AP2/ERF superfamily identified 125 *DlAP2/ERF* genes that were classified into four families, which were further divided into 13 groups with similar motif and structure compositions. The SNPs between the ‘HHZ’ and ‘SJM’ cultivars were diverse and provided valuable insights into the evolutionary characteristics of the longan AP2/ERF superfamily. Some AP2/ERF members may regulate organ development of early SE, seed, root, and flower, and some responded to various hormones, including MeJA, SA, ABA, and 2,4-D. The predicted protein–protein interactions indicate that the Baby Boom (BBM) protein may interact with proteins in the LALF/AGL15 network. The phylogenetic and gene expression analyses provide insights for researching the functions of *AP2/ERF* genes in longan. These findings provide a valuable resource for better understanding the biological roles of individual *AP2/ERF* genes in longan.

## Methods

### Identification and classification of longan AP2/ERF genes

The whole longan genome data were downloaded from the GigaScience DataBase, 2017 [33]. We isolated the AP2/ERF superfamily from the whole genome data as follows. First, we used the HMMER tool to confirm the Pfam number (PF00847) of the AP2 conserved domain. Second, we searched the candidate longan AP2/ERF superfamily members from the Pfam annotation file and extracted the CDS and protein sequences using TBtools [66]. Third, we used the known AP2/ERF sequences as query sequences in BLAST searches (*P* value <1e− 5) against the whole longan genome data to checked if any genes were missing from the existing annotation. Fourth, we used the SMART database (http://smart.embl-heidelberg.de/) to further confirm the presence of the AP2 domain in all members of the DlAP2/ERF superfamily. Redundant sequences were discarded manually. A total of 132 candidate genes corresponding to the AP2/ERF superfamily were obtained. For one incomplete sequence, Dlo_014297.1, which had a 3813-bp N-containing sequence in its CDS sequence and contained only 603 bp, the conserved domain could not be found. So, this sequence and six redundant sequences (Dlo_015114.1, Dlo_010824.1, Dlo_023755.1, Dlo_016792.1, Dlo_014756.1, and Dlo_031620.1) were removed. The genes were named according to the scaffold order of each gene in the genomic dataset. The sequence lengths, molecular weights, and isoelectric points of the corresponding DlAP2/ERF proteins were obtained using ExPasy tools (http://web.expasy.org/protparam/).

### Phylogenetic analysis, sequence analysis, and *cis*-acting elements in the promoters of members of the longan AP2/ERF superfamily

The phylogenetic trees were analyzed using the MEGA 5.0 software with neighbor-joining (NJ) and maximum likelihood (ML) algorithm. The following parameters were used: Poisson model with the NL algorithm, Jones-Taylor-Thornton (JTT) model with the ML algorithm, and pairwise deletion and 1000 bootstrap replications with both algorithms. Although Dlo_007774.3 was identified as an AP2 member by Pfam and BLAST searches, its AP2 domain shared lower similarity than the other AP2 sequences, probably because of sequencing errors in the 3′ end that led to the loss of the AP2 domain in Dlo_007774.3. Therefore, Dlo_007774.3 was not considered and the remaining 124 AP2/ERF members were used to constructed phylogenetic trees. The conserved motifs of the DlAP2/ERF proteins were identified using MEME (http://meme.nbcr.net/meme/intro.html) and were re-edited using the TBtools software. The structure of the *DlAP2/ERF* genes was determined by comparing the CDSs with the general feature format (gff) file using TBtools. We extracted the 2000-bp genomic sequence upstream of the start codon for each *DlAP2/ERF* gene as the putative promoter region, and then used PlantCARE (http://bioinformatics.psb.ugent.be/webtools/plantcare/html/) to predict *cis*-acting elements. We found 116 promoter sequences among the 125 *AP2/ERF* genes in the longan genome dataset, so the promoter sequences of nine genes (Dlo_22310.1, Dlo_22613.1, Dlo_23666.1, Dlo_13163.1, Dlo_025387.1, Dlo_007774.3, Dlo_039227.1, Dlo_032272.1, Dlo_008555.1) were not found. Among the 116 detected promoter sequences, nine (Dlo_025861.1, 1184 bp; Dlo_025226.1, 1879 bp; Dlo_033259.1, 1822 bp; Dlo_026624.11085 bp; Dlo_023524.1, 1004 bp; Dlo_028757.1, 1333 bp; Dlo_030601.1, 1952 bp; Dlo_031619.1, 541 bp; Dlo_002919.1, 1625 bp) that contained > 500 bp but < 2000 bp were included in the *cis*-acting element analysis.

### SNPs, InDels, and AS events analyses of the longan AP2/ERF superfamily

The identified SNPs, InDels, and AS events were extracted from four transcriptome datasets, including three for the ‘Hong He Zi’ (HHZ) cultivar and one for the ‘Si Ji Mi’ (SJM) cultivar. The three ‘HHZ’ transcriptome datasets were the NEC and embryogenic cultures (EC, IcpEC, and GE) dataset, the different light quality (blue-light, white-light, and dark as the control) in ECs dataset, and the hormone (2,4-D and KT) treatments in ECs dataset. The one ‘SJM’ transcriptome dataset was the nine organs (root, stem, leaf, flower bud, flower, young fruit, pericarp, pulp, and seed) dataset. The nine organ ‘SJM’ dataset and different light quality in ECs dataset have been published previously [33, 67]. The NEC and embryogenic cultures (NEC, EC, IcpEC and GE) dataset has been upload to NCBI (SRA accession: PRJNA565345) and the hormone (2,4-D and KT) treatments in ECs dataset is still unpublished. Four synchronized longan NEC and embryogenic cultures (EC, IcpEC, and GE) were obtained as described previously [67–69]. The hormone treatments were as follows: 2,4-D + KT, 2,4-D (1.0 mg/L) and KT (0.5 mg/L) were added to the MS medium; 2,4-D, only 2,4-D (1.0 mg/L) was added to the MS medium; KT, only KT (0.5 mg/L) was added to the MS medium; and MS, no hormone was added to the MS medium. The ECs were cultured in these liquid medium for 24 h. We used GATK [70] to call SNPs and InDels for each sample. It is mainly divided into four steps: First, we used HISAT2 software to mapped the raw RNA-seq reads to reference genome; Then we used Picard software to marked duplicates; Third, splited sequences and performed base recalibration; Finally, we used HaplotypeCaller software to detected SNP and Indel-variant site filtering. GATK Parameters: -allowPotentiallyMisencodedQuals -stand_call_conf 20.0 -stand_emit_conf 20.0 (Detection); −window 35 -cluster 3 -filterName FS -filter“FS > 30.0”-filterName QD -filter“QD < 2.0” (Filter). The SNPs and InDels in the ‘HHZ’ cultivar were collected from more than three samples among every dataset, and the SNPs and InDels in ‘SJM’ cultivar were collected from more than four samples in the dataset.

### Longan AP2/ERF protein interaction networks

The functional protein association networks were constructed using STRING online software (https://string-db.org/) with default parameters on the basis of *Arabidopsis* orthologs.

### Plant materials and treatments

Four synchronized ‘HHZ’ longan NEC and embryogenic cultures (EC, IcpEC, and GE) were obtained as described previously [68, 69]. To investigate the gene expression patterns in response to different hormone treatments, we selected six *DlAP2/ERF* genes for qRT-PCR analysis. The ECs were cultured in Murashige and Skoog (MS) liquid medium supplied with 100 μM MeJA, ABA, GA, or SA for 0, 4, 8, 12, and 24 h. All treated samples were immediately frozen in liquid nitrogen and stored at − 80 °C for subsequent analysis.

### RNA extraction and gene expression analysis

Total RNA was extracted using the TransZol Up reagent kit (TRANS, Beijing, China). The RNA quality was analyzed by agarose gel electrophoresis and quantified using a Nanodrop 2000 spectrophotometer (Thermo Scientific, Wilmington, DE, USA). The RNA was used to synthesize first-strand cDNA using a PrimeScript RT Master Mix (Perfect Real Time) cDNA Synthesis Kit (TaKaRa, Japan). The qRT-PCRs were performed on a Roche Lightcyler 480 instrument using SYBR Green chemistry (Hieff qPCR SYBR Green Master Mix, YEASEN, China). Beta actin (*ACTB*) and eukaryotic elongation factor 1-alpha (*EF-1α*) were used as internal controls. The qRT-PCR was as follows: 95 °C for 30 s, followed by 45 cycles of 95 °C for 10 s and 60 °C for 30 s. Each qRT-PCR was performed in biological triplicates and technical replications. The qRT-PCR data were analyzed using the 2^−△△CT^ method. Details of the primers used in this study were given in Additional file 15: Table S10. The transcriptional analysis data of the nine longan organs (root, stem, leaf, flower bud, flower, pericarp, pulp, young fruit, and seed) were obtained from the ‘SJM’ longan transcriptome dataset, which is deposited in NCBI’s GEO database under accession number GSE84467 [33]. The transcriptional analysis of longan under different light treatments was carried out by Li et al. [67]. The NEC and embryogenic cultures (NEC, EC, IcpEC and GE) dataset has been upload to NCBI (SRA accession: PRJNA565345) and the hormone (2,4-D and KT) treatments in ECs dataset is still unpublished. The hormone treatments were performed in three biological triplicates and the average FPKM values of the three biological triplicates were used to calculate the expression levels of the *DlAP2/ERF* superfamily genes. The transcript abundance of *DlAP2/ERF* genes was calculated as fragments per kilobase of exon model per million mapped reads (FPKM). The thresholds for judging the significance of differential gene expression were FDR ≤0.01 and |log2 (fold change)| ≥1. Heatmaps were constructed using the transformed log2 (FPKM+ 1) values. The hierarchical clustering analysis was performed using Cluster 3.0 software (https://academic.oup.com/).

### Statistical analyses

Each experiment represents three independent biological replicates, and all data are expressed as mean ± standard deviation (SD). Differences among groups were tested using one-way ANOVA and Fisher’s least significant difference test.

## Supplementary information

**Additional file 1: Table S1.** Complete list of *AP2/ERF* genes identified in the *D.longan* genome.

**Additional file 2: Figure S1.** Phylogenetic tree used NJ method representing relationships among AP2 family of longan. The different-colored areas indicate AP2-R1, AP2-R2 and AP2-R3 subgroups respectively.

**Additional file 3: Figure S2.** Phylogenetic tree used ML method representing relationships among AP2/ERF superfamily of longan. The different-curve segment indicate different groups of AP2/ERF superfamily.

**Additional file 4: Table S2.** Details of the *cis*-acting elements identified in this study.

**Additional file 5: Table S3.** The SNP annotation of the DlAP2/ERF superfamily detected in ‘HHZ’ and ‘SJM’ clutivars. a The SNP annotation of the DlAP2/ERF superfamily detected in the NEC and embryogenic cultures (EC, IcpEC and GE) of ‘HHZ’ cultivar. b The SNP annotation of the DlAP2/ERF superfamily detected in the EC under light quality (blue, white, and dark as the control) treatments. c The SNP annotation of the DlAP2/ERF superfamily detected in the EC under hormone (2,4-D and KT) treatments. d The SNP annotation of the DlAP2/ERF superfamily detected in nine organs of ‘SJM’ cultivar.

**Additional file 6: Table S4.** The InDel annotation of the DlAP2/ERF superfamily detected in ‘HHZ’ and ‘SJM’ clutivars. a The InDel annotation of the DlAP2/ERF superfamily detected in the NEC and embryogenic cultures of ‘HHZ’ cultivar. b The InDel annotation of the DlAP2/ERF superfamily detected in the EC under light quality treatments. c The InDel annotation of the DlAP2/ERF superfamily detected in the EC under hormone treatments. d The InDel annotation of the DlAP2/ERF superfamily detected in nine organs of ‘SJM’ cultivar.

**Additional file 7: Table S5.** The alternative splicing events of the DlAP2/ERF superfamily detected in the NEC and embryogenic cultures of longan. a The alternative splicing events (alternative 3′ splice site acceptor) of the DlAP2/ERF superfamily detected in the NEC and embryogenic cultures of ‘HHZ’ cultivar. b The alternative splicing events (5′ splice site donor) of the DlAP2/ERF superfamily detected in the NEC and embryogenic cultures of ‘HHZ’ cultivar. c The alternative splicing events (intron retention) of the DlAP2/ERF superfamily detected in the NEC and embryogenic cultures of ‘HHZ’ cultivar. d The alternative splicing events (exon skipping) of the DlAP2/ERF superfamily detected in the NEC and embryogenic cultures of ‘HHZ’ cultivar.

**Additional file 8: Table S6.** The alternative splicing events of the DlAP2/ERF superfamily detected in the EC under light quality treatments. a The alternative splicing events (alternative 3′ splice site acceptor) of the DlAP2/ERF superfamily detected in the EC under light quality treatments. b The alternative splicing events (5′ splice site donor) of the DlAP2/ERF superfamily detected in the EC under light quality treatments. c The alternative splicing events (intron retention) of the DlAP2/ERF superfamily detected in the EC under light quality treatments. d The alternative splicing events (exon skipping) of the DlAP2/ERF superfamily detected in the EC under light quality treatments.

**Additional file 9: Table S7.** The alternative splicing events of the DlAP2/ERF superfamily detected in the EC under hormone treatments. a The alternative splicing events (alternative 3′ splice site acceptor) of the DlAP2/ERF superfamily detected in the EC under hormone treatments. b The alternative splicing events (5′ splice site donor) of the DlAP2/ERF superfamily detected in the EC under hormone treatments. c The alternative splicing events (intron retention) of the DlAP2/ERF superfamily detected in the EC under hormone treatments. d The alternative splicing events (exon skipping) of the DlAP2/ERF superfamily detected in the EC under hormone treatments.

**Additional file 10: Table S8.** The alternative splicing events of the DlAP2/ERF superfamily detected in nine organs of ‘SJM’ cultivars. a The alternative splicing events (alternative 3′ splice site acceptor) of the DlAP2/ERF superfamily detected in nine organs of ‘SJM’ cultivars. b The alternative splicing events (5′ splice site donor) of the DlAP2/ERF superfamily detected in nine organs of ‘SJM’ cultivars. c The alternative splicing events (intron retention) of the DlAP2/ERF superfamily detected in nine organs of ‘SJM’ cultivars. d The alternative splicing events (exon skipping) of the DlAP2/ERF superfamily detected in nine organs of ‘SJM’ cultivars.

**Additional file 11: Table S9.** RNA-seq data and differentially expressed *DlAP2/ERF* genes used in this study. a RNA-seq data of the DlAP2/ERF superfamily used in this study. b Differentially expressed *DlAP2/ERF* genes in different development stages and treatments.

**Additional file 12: Figure S3.** Hierachical clustering of expression profiles of longan *AP2/ERF* genes in different light quality treatment.

**Additional file 13: Figure S4.** Hierachical clustering of expression profiles of longan *AP2/ERF* genes in different hormone treatment. X-axis labels are the same as Fig. 6.

**Additional file 14: Figure S5.** Hierachical clustering of expression profiles of longan *AP2/ERF* genes in nine organs of ‘SJM’ cultivar.

**Additional file 15: Table S10.** Primers used in quantitative RT-PCR of *DlAP2/ERF* genes.

**Additional file 16: Table S11.** The information for each motif of DlAP2/ERFs.

## Data Availability

All data presented in this study are provided either in the manuscript or additional files.

## Abbreviations

2,4-D: 2,4-Dichlorophenoxyacetic acid
aa: amino acid
ABA: Abscisic acid
AP2/ERF: APETALA2/ethylene responsive factor
AS: Alternative splicing
BBM: Baby Boom
CDS: Coding sequence
EC: Embryogenic callus
FPKM: Fragments per kilobase of exon per million fragments
GA: Gibberellin
GE: Globular embryos
IcpEC: Incomplete compact proembryogenic cultures
InDels: Insertions and deletions
KT: Kinetin
MeJA: Methyl jasmonate
MS: Murashige and Skoog
MW: Molecular weight
NEC: Non-embryogenic callus
pI: Isoelectric point
QRT-PCR: Quantitative real-time polymerase chain reaction
SA: Salicylic acid
SE: Somatic embryogenesis
SNPs: Single nucleotide polymorphisms
