# Supplementary figures and images for: Genome-wide identification, molecular evolution, and expression analysis provide new insights into the APETALA2/ethylene responsive factor (AP2/ERF) superfamily in Dimocarpus longan Lour
Source: BMC Genomics. 2020 Jan 20;21:62. doi: 10.1186/s12864-020-6469-4 (PMC6971931; doi:10.1186/s12864-020-6469-4)

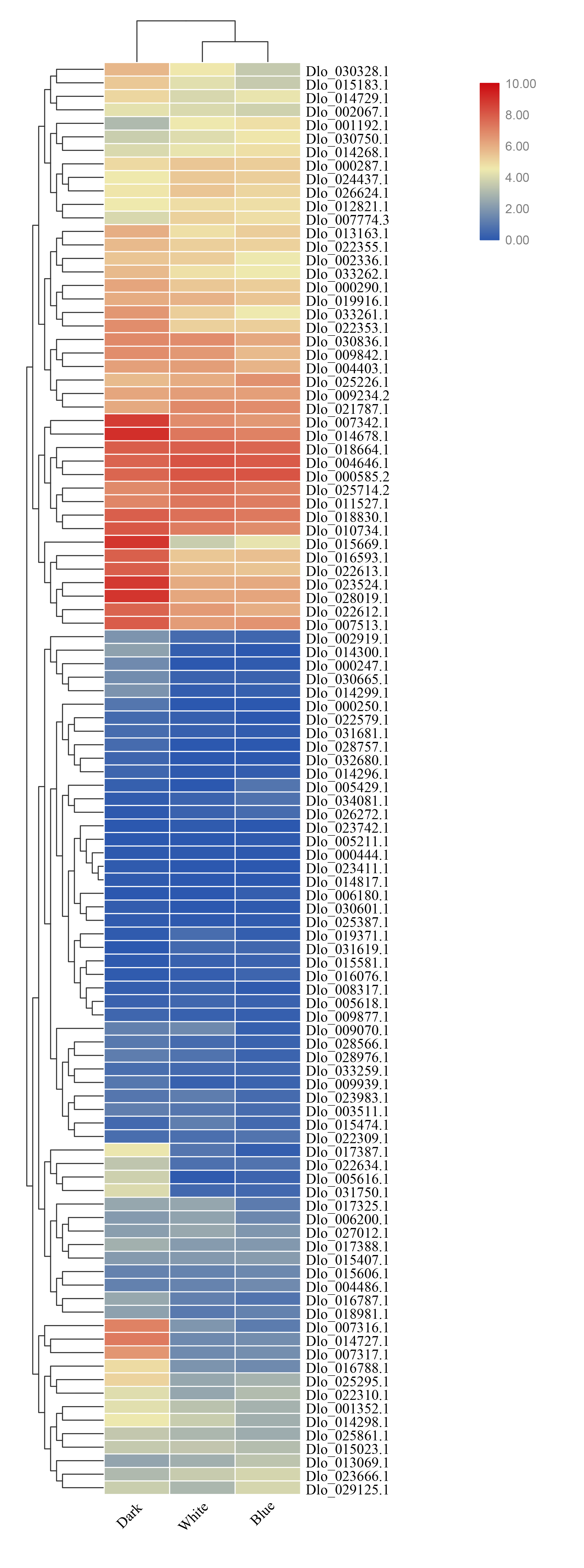

Supplement: Supplementary file 4 — Additional file 4: Table S2. Details of the cis-acting elements identified in this study. [file 12864_2020_6469_MOESM4_ESM.tif]

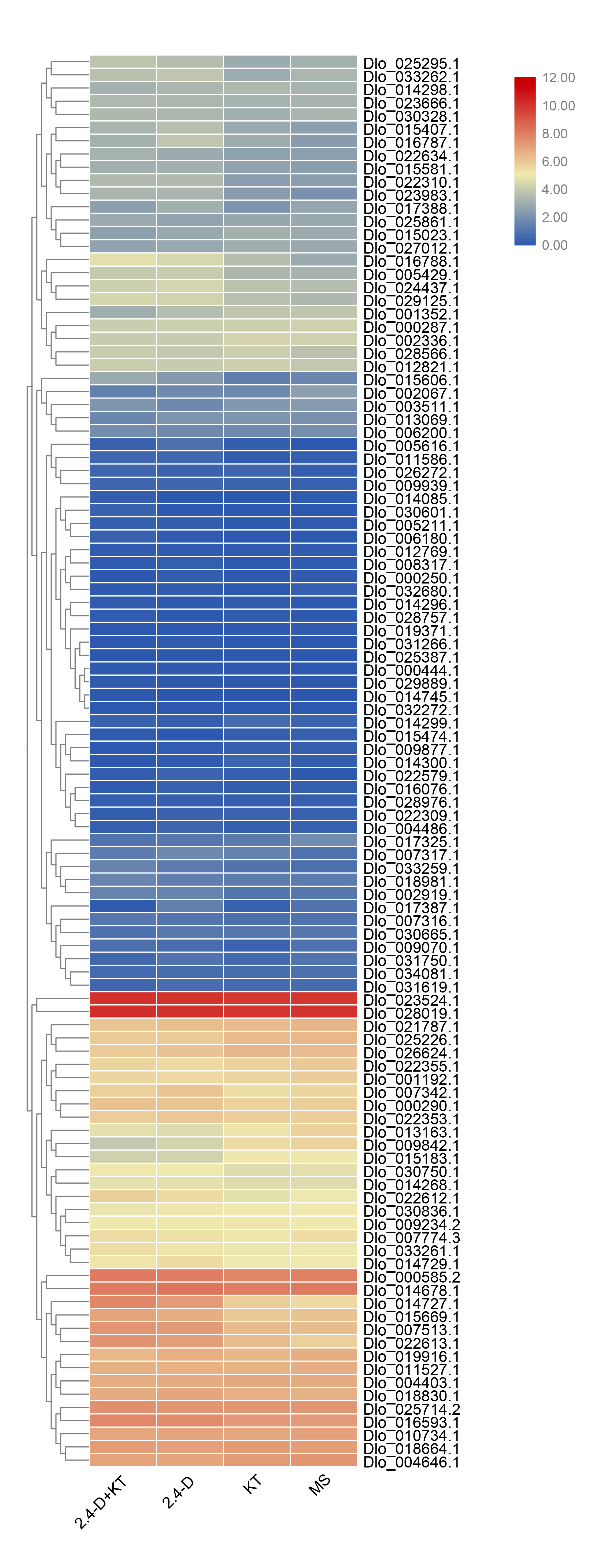

Supplement: Supplementary file 5 — Additional file 5: Table S3. The SNP annotation of the DlAP2/ERF superfamily detected in ‘HHZ’ and ‘SJM’ clutivars. a The SNP annotation of the DlAP2/ERF superfamily detected in the NEC and embryogenic cultures (EC, IcpEC and GE) of ‘HHZ’ cultivar. b The SNP annotation of the DlAP2/ERF superfamily detected in the EC under light quality (blue, white, and dark as the control) treatments. c The SNP annotation of the DlAP2/ERF superfamily detected in the EC under hormone (2,4-D and KT) treatments. d The SNP annotation of the DlAP2/ERF superfamily detected in nine organs of ‘SJM’ cultivar. [file 12864_2020_6469_MOESM5_ESM.tif]

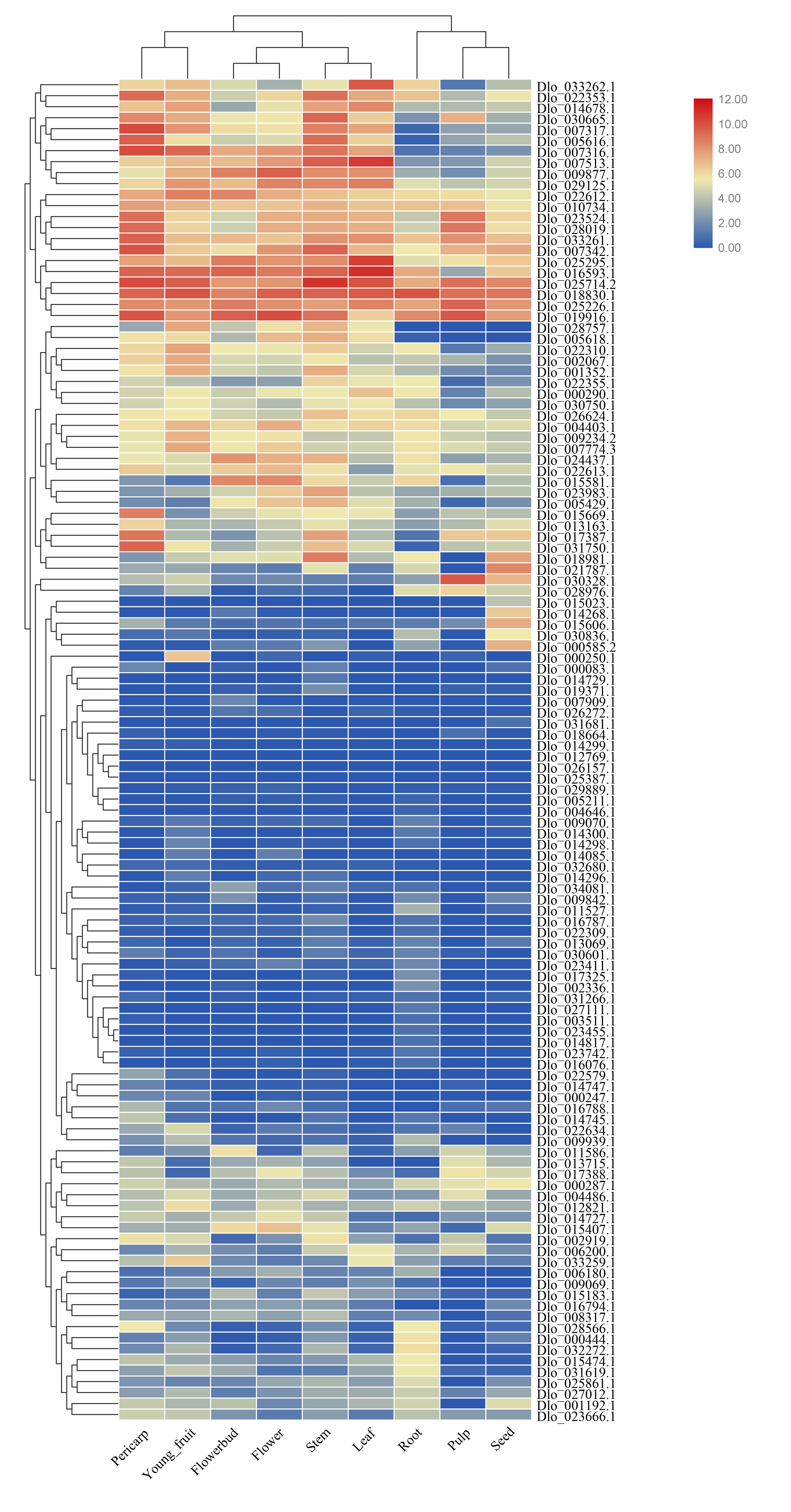

Supplement: Supplementary file 6 — Additional file 6: Table S4. The InDel annotation of the DlAP2/ERF superfamily detected in ‘HHZ’ and ‘SJM’ clutivars. a The InDel annotation of the DlAP2/ERF superfamily detected in the NEC and embryogenic cultures of ‘HHZ’ cultivar. b The InDel annotation of the DlAP2/ERF superfamily detected in the EC under light quality treatments. c The InDel annotation of the DlAP2/ERF superfamily detected in the EC under hormone treatments. d The InDel annotation of the DlAP2/ERF superfamily detected in nine organs of ‘SJM’ cultivar. [file 12864_2020_6469_MOESM6_ESM.tif]

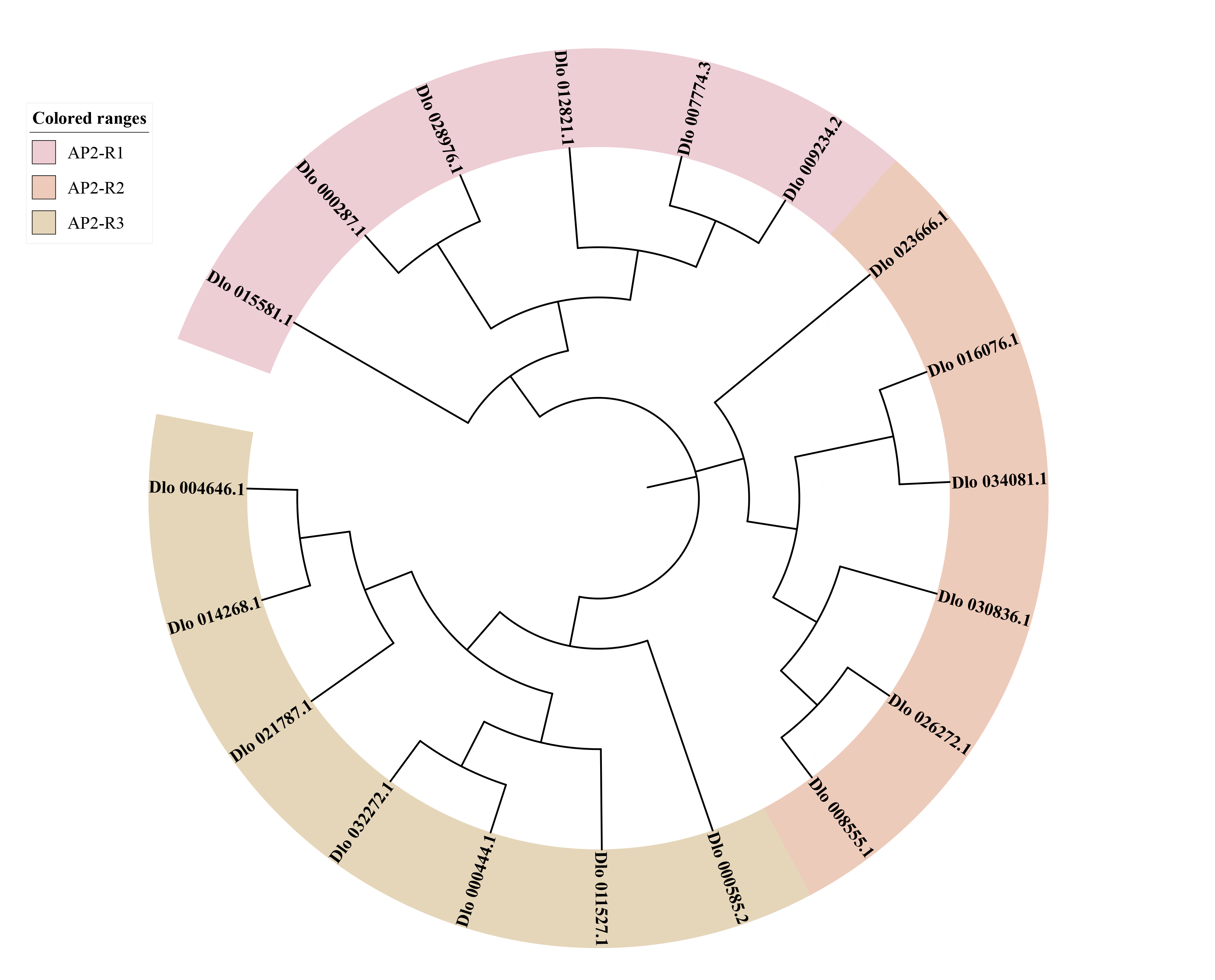

Supplement: Supplementary file 9 — Additional file 9: Table S7. The alternative splicing events of the DlAP2/ERF superfamily detected in the EC under hormone treatments. a The alternative splicing events (alternative 3′ splice site acceptor) of the DlAP2/ERF superfamily detected in the EC under hormone treatments. b The alternative splicing events (5′ splice site donor) of the DlAP2/ERF superfamily detected in the EC under hormone treatments. c The alternative splicing events (intron retention) of the DlAP2/ERF superfamily detected in the EC under hormone treatments. d The alternative splicing events (exon skipping) of the DlAP2/ERF superfamily detected in the EC under hormone treatments. [file 12864_2020_6469_MOESM9_ESM.tif]

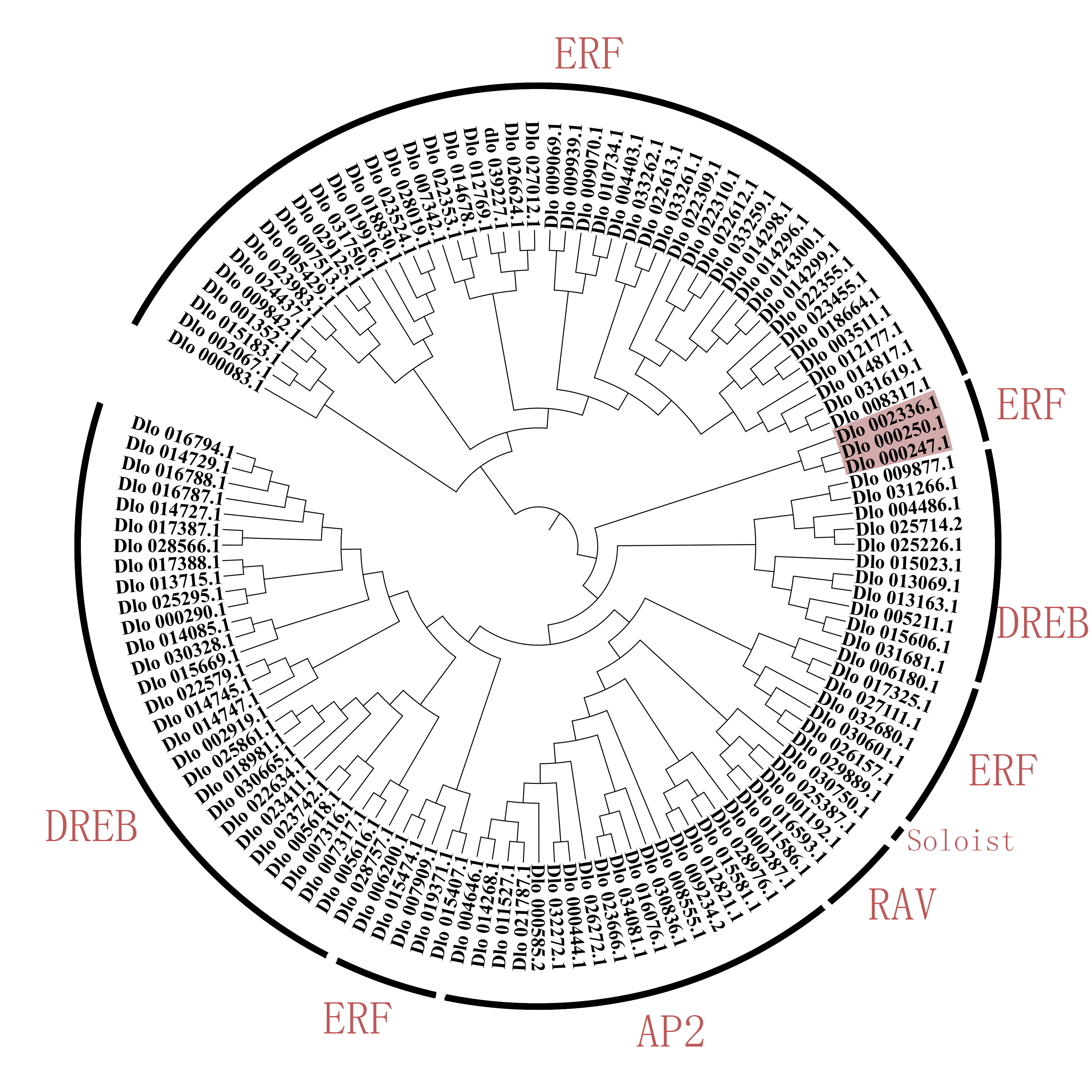

Supplement: Supplementary file 10 — Additional file 10: Table S8. The alternative splicing events of the DlAP2/ERF superfamily detected in nine organs of ‘SJM’ cultivars. a The alternative splicing events (alternative 3′ splice site acceptor) of the DlAP2/ERF superfamily detected in nine organs of ‘SJM’ cultivars. b The alternative splicing events (5′ splice site donor) of the DlAP2/ERF superfamily detected in nine organs of ‘SJM’ cultivars. c The alternative splicing events (intron retention) of the DlAP2/ERF superfamily detected in nine organs of ‘SJM’ cultivars. d The alternative splicing events (exon skipping) of the DlAP2/ERF superfamily detected in nine organs of ‘SJM’ cultivars. [file 12864_2020_6469_MOESM10_ESM.tif]
